# Developing conceptions of forgiveness across the lifespan

**DOI:** 10.1111/cdev.14122

**Published:** 2024-05-31

**Authors:** Abby McLaughlin, Julia Marshall, Katherine McAuliffe

**Affiliations:** ^1^ Department of Psychology and Neuroscience Boston College Chestnut Hill Massachusetts USA

## Abstract

Understanding how to respond to transgressions is central to cooperation, yet little is known about how individuals understand the consequences of these responses. Accordingly, the current study explored children's (ages 5–9), adolescents' (ages 11–14), and adults' (*N* = 544, predominantly White, ~50% female, tested in 2021) understandings of three such responses—forgiveness, punishment, and doing nothing. At all ages, participants differentiated between the consequences of these three responses. Forgiveness was associated with more positive and fewer negative outcomes, while the opposite was true for punishment and doing nothing. With age, participants were less likely to expect positive outcomes, and this effect was strongest for punishment and doing nothing. The results of this study allow novel insights into reasoning about three important response strategies.

**Abbreviations**
ANOVA: analysis of variance
CLRN: Character Lab Research Network

## INTRODUCTION

Interpersonal conflict is an inevitable part of social relationships in large‐scale human cooperative societies. As members of cooperative groups, we often hold conflicting goals and act to maximize our own desired outcomes at others' expense. The universality of conflict requires that people develop a toolkit of solutions for responding to interpersonal transgressions, mediating disputes, and resolving conflicts. This is particularly important when they themselves are the victims of transgressions. Two of these strategies—and those on which we focus in the present study—are punishment and forgiveness. We explore conceptions of punishment and forgiveness in children, adolescents, and adults to understand whether and to what extent intuitions about the consequences of punishment and forgiveness evolve across ages. We compare conceptions of these two responses to a third alternative, which is doing nothing in response to a transgression. Although these three response strategies do not represent an exhaustive set of potential responses, studying forgiveness and punishment allows us to explore two valued, commonly enacted responses that are often viewed as essential to the pursuit of justice.

While previous research has provided some insight into how children and adults understand and engage in punishment (reviewed in Marshall & McAuliffe, 2022) and forgiveness (reviewed in Van der Wal et al., 2016) separately, the current study has the potential to expand our understanding of how individuals differentiate between these responses when they are considered together. By investigating the consequences that children, adolescents, and adults expect to follow forgiveness, punishment, or doing nothing, we can gain insight into developmental changes and the motives that may underlie decisions to enact particular responses following a transgression.

### Punishment

Punishment, operationally defined as the imposition of a penalty in response to a transgression (e.g., Clutton‐Brock & Parker, 1995; Raihani et al., 2012), is observed early in development (Marshall & McAuliffe, 2022) and across cultures (Henrich et al., 2006). Evolutionary perspectives have argued that punishment functions to ensure cooperation among non‐kin and in larger‐scale societies where reputation alone cannot discourage antisocial behavior (Henrich et al., 2006). Research on punishment has typically differentiated between second‐party punishment, which is punishment meted out by the victim of a transgression, and third‐party punishment, which is done by an uninvolved witness to the transgression; the current paper will focus only on second‐party cases. Focusing only on second‐party punishment in the present study allows us to home in on victims' responses to interpersonal transgressions and to make more direct comparisons between punishment and forgiveness. Second‐party punishment decisions have been studied with adult participants through economic games, such as the Ultimatum Game (e.g., Gülf et al., 1982), as well as in evaluative vignette studies (e.g., Martin et al., 2019) and behavioral studies approximating real‐world conditions (e.g., Gollwitzer et al., 2011). This body of work indicates that adults readily engage in second‐party punishment, even when doing so is costly. Adults display behavior consistent with a variety of motives—including creating fair outcomes (i.e., Deutchman et al., 2020), reciprocating harms (i.e., Tonry, 2011), and teaching lessons (i.e., Crockett et al., 2014; Solomon & Murphy, 1990)—which can provide insight into the outcomes expected in the aftermath of punishment.

From a developmental perspective, children are motivated to punish when they are victims of transgressions (Bereby‐Meyer & Fiks, 2013; Gummerum & Chu, 2014). Previous research has documented children's punitive behavior experimentally, in the context of economic games, and in real‐life settings through observational studies. In the domain of economic games, studies have demonstrated that children, when faced with unfairness in an Ultimatum Game, are apt to punish the perpetrators of this unfairness (McAuliffe & Dunham, 2017; Sutter, 2007). Starting at 5 years of age, children will punish others who treat them unfairly, even when punishment comes at a cost (Wittig et al., 2013). Observational data indicates that children enact punishment in the real world (Caporaso & Marcovitch, 2021; Zahn‐Waxler et al., 1996), implementing strategies varying from aggressive behavior to tattling.

While we know from this previous research that children engage in second‐party punishment in natural and experimental settings, we know little about what they expect will happen as a consequence. The lack of insight from existing work into children's expectations following intervention makes it difficult to understand what factors children may be taking into account as they weigh their decisions to punish or enact an alternative response. Some work suggests that even though children punish when they are victims of a transgression, they negatively evaluate second‐party punishment (Strauß & Bondü, 2022). This tension makes it particularly critical and interesting to explore what children expect to occur in the aftermath of punishment and to directly compare these expectations with those associated with forgiveness or doing nothing. In the current study, we explore the behavioral and affective outcomes associated with second‐party punishment across the lifespan in order to gain insight into the nuances of how individuals perceive this response strategy as well as how it may compare to other responses.

### Forgiveness

Forgiveness has been defined by psychologists in many different ways: as “a suite of prosocial motivational changes that occurs after a person has incurred a transgression” (McCullough, 2001, p. 194) or as the process of “[giving] up their right to resentment and [offering] kindness, respect, generosity, and even love to the one or ones who acted unfairly” (Enright & Song, 2020). Enright et al. (1989) present a theory of developmental stages of forgiveness, with six stages parallel to but distinct from Kohlberg's (1958) moral developmental stages, in which individuals progress from viewing forgiveness as contingent on revenge to viewing forgiveness as motivated by love. In their seminal work on the psychology of forgiveness, Worthington and colleagues introduced a distinction between decisional and emotional forgiveness, defining decisional forgiveness as “a change in a person's behavioral intentions … toward a transgressor” and emotional forgiveness as “a replacement of negative, unforgiving emotions with positive, other‐oriented emotions” (reviewed in Worthington, 2005, p. 4).

Regardless of the particular definition being used, forgiveness is understood to serve an important role in repairing damaged relationships and restoring intragroup harmony in the aftermath of interpersonal transgressions. Psychologists have argued that forgiveness may have emerged in humans to mend social relationships when the transgressor is socially valuable and has shown that there is low risk of future exploitation, thereby improving the victim's long‐term welfare (e.g., McCauley et al., 2022). Forgiveness is generally viewed by adults as a prosocial response to transgressions, although individual‐level differences such as agreeableness, emotional stability, and religiosity have been shown to predict evaluations of forgiveness (McCullough, 2001). Much research has been conducted on adults' perceptions of the consequences of forgiveness, with a focus on relationships, risks of re‐offense, and expectations of future interactions between offender and victim (e.g., Strelan et al., 2017; Wallace et al., 2008). These studies suggest that adults tend to be wary of the negative consequences that may arise after forgiveness and often take these concerns into account when deciding whether to forgive an offender.

While forgiveness in adults has received much attention from psychologists in recent years, research on children's forgiveness—a growing area (e.g., Rapp et al., 2022) with deep roots in our field (e.g., Enright, 1994; Enright et al., 1989)—has received relatively less empirical attention. Initial evidence suggests that, by age four, children engage in forgiveness in experimental paradigms (Oostenbroek & Vaish, 2019a, 2019b). Four‐year‐olds are more likely to share resources with and positively rate transgressors who say sorry than transgressors who do not (Oostenbroek & Vaish, 2019a, 2019b). Similarly, 5‐year‐olds prefer transgressors who display guilt through facial expressions (Drell & Jaswal, 2016) to those who do not display guilt. By mid‐childhood, children show sensitivity to intentionality—children are more likely to return valued resources to an actor who accidentally, versus intentionally, committed an antisocial act (Amir et al., 2021; McElroy et al., 2023). Starting at age nine, according to work published by Enright et al. (1989), children make sophisticated judgments about the conditions that must be in place for forgiveness to occur, often referring to punishment, revenge, or offender reparations. In this (Enright et al., 1989) and related studies (e.g., Enright, 1994; Huang & Enright, 2000), researchers provide evidence for a stage model of forgiveness development that parallels Kohlberg's model of moral development, moving from forgiveness as requiring vengeance to viewing forgiveness as an act of love. Findings from these lines of work raise the possibility that children forgive because they expect forgiveness to generate positive consequences or because they feel that particular conditions have been met by perpetrators. However, based on existing evidence, we cannot directly address these possibilities.

In addition to the work conducted on children's tendencies to engage in forgiveness behavior as victims, some previous research has also documented children's evaluations of forgiving victims. For instance, in a study by Oostenbroek and Vaish (2019a, 2019b), children who observed accidental transgressions between two adults shared more with and reported greater liking for forgiving victims than unforgiving victims. This study suggests that by age five, children value those who engage in forgiveness. A related study found that young children associate forgiveness with positive outcomes (such as smiling and reinitiating play), providing additional evidence for the idea that, by the preschool years, children perceive forgiveness positively (Ahirwar et al., 2019). Although these studies tell us that, in their earliest years, children are willing to forgive transgressions and view forgiveness as a prosocial response, we still do not have a robust understanding of the ways in which children are reasoning about forgiveness.

### Doing nothing

Instead of punishing or forgiving in the face of a transgression, victims may choose to do nothing. Although this may not be considered a response per se, we believe understanding intuitions surrounding doing nothing is important because it is a readily available reaction to an interpersonal transgression that involves some behavioral aspects of forgiveness (i.e., choosing not to punish) but does not necessarily involve a motivational change. Refraining from punishment could be seen as indicative of forgiveness, yet the decision to avoid punishment may not signify actual attitudinal shifts in the victim. Likewise, the decision to refrain from offering forgiveness should not be conflated with punishment, as the victim's unwillingness to excuse the offender does not mean that they necessarily seek to impose a cost. Inaction clearly exists as a third category distinct from punishment and forgiveness, and thus it is critical to explore how the consequences associated with this response compare to more frequently studied responses. Moreover, including doing nothing as a condition allows us to address the difference in behavioral versus intrapersonal forms of punishment and forgiveness. By asking participants what outcomes are expected after a decision to do nothing, we can make more direct comparisons between these three options as well as determine how doing nothing may pattern differently than punishment or forgiveness.

While previous literature has tended to independently explore evaluations of and engagement in forgiveness (e.g., Oostenbroek & Vaish, 2019a, 2019b) and punishment (as reviewed in Marshall & McAuliffe, 2022), there exists a considerable gap in our understanding of how individuals reason about the consequences of these response strategies. In particular, by failing to make direct comparisons between evaluations of forgiveness, punishment, and doing nothing, the previous literature ignores potential similarities and differences between them. Accordingly, the present study examines children's, adolescents', and adults' perceptions of the consequences of forgiveness, punishment, and doing nothing by asking participants to rate the likelihood of various behavioral and affective outcomes. In doing so, we hope to address three main questions, which we will introduce below, regarding the development of forgiveness‐ and punishment‐related judgments. These three research questions are intended to serve as the foundation for the present investigation. Although we speculate about possible answers to these questions, it is important to acknowledge that the majority of this work is exploratory and is intended to shed initial light on questions surrounding the development of conceptualizations of punishment, forgiveness, and doing nothing. For this reason, we do not log specific directional hypotheses.

First, to what extent are forgiveness, punishment, and doing nothing viewed as conceptually distinct in terms of their consequences? Although previous research has documented children's tendency to enact both punishment (Lamb et al., 1980) and forgiveness (Amir et al., 2021; Enright et al., 1989; Oostenbroek & Vaish, 2019a, 2019b) in experimental and real‐world contexts, and Enright et al. (1989) explored concepts of forgiveness in relation to moral development more broadly, it remains to be explored whether children distinguish between the consequences of these intervention strategies. It may be the case that, early in development, children do not make distinctions between the outcomes associated with forgiveness, punishment, and doing nothing and instead view all interventions as leading to similar consequences. This would align with Enright et al.'s (1989) view of the early stages of forgiveness as involving retributive motives and may suggest that children do not actually reason about forgiveness and punishment in sophisticated ways. In contrast, it may be possible that children do distinguish between these three intervention strategies and thus hold nuanced beliefs about what will occur after a victim has been forgiven, punished, or decided to do nothing. Research in developmental and moral psychology provides some evidence that children hold sophisticated beliefs about both forgiveness and punishment, but the present study will be among the first to compare these response strategies in a single paradigm.

Second, how do the perceived consequences of forgiveness, punishment, and doing nothing compare to one another? As previously mentioned, some definitions of forgiveness seem to interpret a lack of punitive behavior (i.e., doing nothing) as conferring forgiveness on transgressors (e.g., McCullough, 2001). This definition does not align with the multidimensional theory of forgiveness, which suggests that forgiveness consists of a behavioral component (i.e., refraining from punishment) and an affective component (i.e., shift from negative to positive other‐oriented emotions) (e.g., Thompson et al., 2005). The present study has the potential to contribute to a greater understanding of how forgiveness, punishment, and doing nothing  may lead to similar or different behavioral, affective, and evaluative consequences and thus shed light on why individuals may choose a specific response in a given situation. We have reason to expect that participants, especially younger participants, may hold stronger intuitions regarding the consequences of punishment compared to forgiveness. Previous research documenting children's real‐world behavior and experiences (e.g., Caporaso & Marcovitch, 2021; Recchia et al., 2019) suggests that, at an early age, children frequently experience and witness norm enforcement, including punishment, which may then lead them to hold more complex beliefs about what occurs in its aftermath. Moreover, punishment has observable behavioral consequences (e.g., timeouts, loss of privileges or desired resources), whereas forgiveness is often viewed as an internal process, meaning that children may be less aware of or attentive to the process of forgiveness when it does occur in their social environments.

Third, how do perceptions of forgiveness, punishment, and doing nothing change over development? While existing lines of work have explored forgiveness and punishment in young children and adults independently, relatively few studies have used the same methods across age groups to assess these questions, making it difficult to clarify developmental trajectories (but see Dunlea & Heiphetz, 2021; Gummerum et al., 2020; Sutter, 2007 for notable exceptions). Previous studies that have tested both children and adults have found generally consistent patterns in punishment behavior and evaluations, but this body of work has also found some developmental differences. For example, young children are more likely than adults to conceptualize peer punishment as driven by positive motivations, such as reforming a transgressor's behavior (Marshall et al., 2021), and are also more likely than adults to view punishment as a means of redemption (Dunlea & Heiphetz, 2021). In the domain of forgiveness, even fewer studies have compared young children and adults, and thus there remain many open questions regarding how evaluations of and engagement in forgiveness may shift over development. A 2022 meta‐analysis from Rapp and colleagues found that educational interventions are effective in promoting forgiveness in participants as young as 6 years of age and throughout adolescence, but this line of work still neglects the conceptions of forgiveness across a broad age range. As individuals encounter increasing incidents of interpersonal conflict and various interventions over their lifespan, we may expect that older participants will be more likely to differentiate between the consequences of various interventions. Because we see increasingly sophisticated judgments about forgiveness over the course of development, older participants may be less likely than younger participants to conflate outcomes associated with forgiveness and doing nothing.

Moreover, research in other domains suggests that over the course of development, people become increasingly able to form multi‐dimensional, rather than dichotomous, views of justice and related concepts. We know from previous work from Huppert et al. (2019) that children across societies initially show strong attitudes about fairness and negatively evaluate any allocations that do not adhere to principles of equality. Throughout early childhood, however, children become more aware of factors such as merit that may justify unequal distributions, thus indicating greater nuance in their principles (Huppert et al., 2019). Similarly, classical studies from Kohlberg (1958) show a shift from a strict adherence to rules to an acknowledgment of complexity and a weighing of personal ethics. A similar shift may reveal itself in developmental changes in reasoning about forgiveness and punishment. Compared to adolescents and adults, who may hold nuanced theories about the consequences of different interventions, children may be more likely to view forgiveness as wholly good and punishment as wholly bad.

Beyond this explanation, there are also numerous cognitive changes over the lifespan, due to which we may expect changes in the perceptions of these response strategies. In particular, Theory of Mind, the ability to understand others' mental states, may influence perceptions of the emotional consequences of different responses. Based on research using real‐apparent emotion tasks (e.g., Harris et al., 1986), we expect that the ability to understand hidden emotions will be lower among child participants compared to adolescents and adults, and this ability may be implicated in questions about emotional responses to transgressions and various response strategies. This would lead children to be less likely to differentiate between the outcomes associated with different response strategies than adolescents or adults. Other cognitive abilities, such as cognitive flexibility (e.g., Wang et al., 2021) and emotional comprehension (e.g., Pons et al., 2004), may facilitate the emergence of increasingly nuanced and sophisticated views of forgiveness and punishment with age.

To better understand how people reason about the consequences of forgiveness, punishment, and doing nothing, we generated a set of 12 “consequence” items, representing outcomes participants may expect to occur in the aftermath of these response strategies (see Table 1 for items and related references). Note that, although we asked participants what they expected to happen *after* a victim forgave, we did not intend to present forgiveness as a dichotomous response and agree with conceptualizations of forgiveness as a process, as described above. Although this set of items was not intended to be exhaustive, they were created by extracting relevant themes from existing literature with the aim of capturing important potential distinctions and similarities between the three response types. These 12 consequence items are presented in four conceptual categories—positive behaviors, negative behaviors, affective change, and affiliative interest—to provide an overarching framework for our hypotheses and results. These category labels are meant to structure our hypotheses and results but are not meant to capture naturalistic groupings of consequence items and are thus not used to collapse across items for analyses. By examining how children, adolescents, and adults reason about these items, we aimed to shed light on how individuals conceptualize the consequences of different interventions across development.

**TABLE 1:** Dependent variables, verbatim question text, and relevant citations for 12 consequence items included in study design.

Dependent variable (consequence item)	Question text	Relevant citations
Positive behaviors		
Victim's Future Trust of Offender	Do you think [victim] will trust [offender] to watch their [scooter/laptop] when they go inside?	van der Wal et al. (2016)
Victim's Prosocial Thoughts about the Offender	Do you think [victim] will think nice things about [offender]?	McCullough (2001) Oostenbroek & Vaish (2019a)
Victim's Empathy for the Offender	Do you think [victim] will try to think about how [offender] feels?	Zechmeister & Romero (2002)
Negative behaviors		
Victim's Avoidance of Offender	Do you think [victim] will try to avoid [offender]?	Oostenbroek & Vaish (2019a) Strelan (2018) Gromet & Okimoto (2014)
Victim's Willingness to Gossip about Offender	Do you think [victim] will say mean things about [offender] to the other [kids at school/people at work]?	Recchia et al. (2019)
Victim's Pursuit of Revenge	Do you think [victim] will try to get back at [offender] for what they did?	Smith & Warneken, 2016 Recchia et al. (2019)
Offender Recidivism	Do you think that [offender] will [commit the same violation] again in the future?	Bregant et al. (2016)
Affective change		
Victim's Positive Affect	Do you think [victim] will feel calm and happy?	van der Wal et al. (2016) Strelan et al. (2017) Strelan et al. (2020)
Offender's Positive Affect	Do you think [offender] will feel calm and happy?	
Bystander's Positive Affect	Do you think the other [kids at school/people at work] will feel safe and happy?	Bregant et al. (2016) Okimoto & Wenzel (2009)
Affiliative interest		
Others' Interest in Affiliating with the Victim	Do you think the other [kids at school/people at work] will want to [play/work] with [victim]?	Oostenbroek & Vaish (2019a)
Others' Interest in Affiliating with the Offender	Do you think the other [kids at school/people at work] will want to [play/work] with [offender]?	Smith et al. (2010) Yao & Chao (2019)

*Note*: Relevant citations provided theoretical grounding for consequence items and question text but did not include any of the items verbatim.

There remains much to be known about how individuals conceive of forgiveness and punishment, how such concepts differ from one another and from doing nothing, and how such conceptions develop from childhood into adulthood. The current project aims to address this gap in the literature by directly comparing how children, adolescents, and adults perceive the consequences of forgiveness, punishment, and doing nothing. By making these direct comparisons, the present study can shed light on what individuals expect to occur after these interventions and the extent to which, across the lifespan, people are discriminating between the outcomes associated with each.

## METHODS

### Design

The study design included two within‐subject factors—condition (forgive, punish, and do nothing) and story (bike, basketball, and drawing)—and one between‐subjects factor—age group (children, adolescents, and adults), meaning it was a 3 by 3 by 2 mixed factorial design. All participants were tested in accordance with our IRB approved research protocol at Boston College; Protocol #: 16.242.01. We began data collection on June 29, 2021.

### Participants

Our child sample consisted of 129 children between the ages of 5 and 9 (*M*_age_ = 7.38, SD_age_ = 1.56, min = 5, max = 9) [https://aspredicted.org/blind.php?x=HBJ_3NB]. We chose this age range because children reliably engage in both forgiveness (Oostenbroek & Vaish, 2019a, 2019b) and second‐party punishment by 5 years of age (Wittig et al., 2013) and show punishment in response to transgression later in development (Gummerum & Chu, 2014; Sutter, 2007). Our preregistered target of *N* = 130 provided us with enough power (*α* = .05) to detect a medium‐sized effect of Condition (Cohen's *d* = .50) and associated pairwise comparisons and with 80% power (*α* = .05) to detect a medium‐sized interaction (Cohen's *d*_z_ = .50) between Age and Condition and associated pairwise comparisons. We collected data from a total of 133 participants and excluded 4 of these participants due to disclosed diagnoses of developmental disorders (*n* = 1, pre‐registered criterion), experimenter error (*n* = 2, pre‐registered criterion), or lack of fluency in English (*n* = 1, not pre‐registered criterion), leaving a final sample of 129 participants. Our final sample was 43% female and 57% male and was predominantly White (45.7%) with the remaining participants reporting their race and ethnicity as Asian or Asian American (12.4%), Black or African American (3.9%), Hispanic or Latino (3.9%), or multiracial (20.9%), with 3.1% choosing not to report racial and ethnic information. Participants were recruited from an existing database and through general laboratory advertisements posted on social media platforms. Parents who signed up for the lab database were contacted about participation via email, and children were tested over Zoom.

Our adolescent sample was tested in collaboration with the Character Lab Research Network (CLRN), a nonprofit organization that works to facilitate research on topics related to youth development (Character Lab Research Network, n.d.) [https://aspredicted.org/blind.php?x=7Z1_8XV]. We requested a target sample of 200 participants between the ages of 11 and 14, and our final sample consisted of 217 adolescents who completed the entire survey (*M*_age_ = 12.68, SD_age_ = 1.18, min = 11, max = 15). Our sample was 41% female, 48% male, and 4% other, with 7% not reporting gender, and was 72% White, 16% Black, 6% Asian, and 45% Hispanic. 119 participants were excluded for not completing the study.

Our adult sample consisted of 198 participants (*M*_age_ = 39.53, SD_age_ = 10.48, min = 21, max = 66) tested on CloudResearch's MTurk and included 93 females, 103 males, and 1 non‐binary participant, with one participant not reporting gender. [https://aspredicted.org/blind.php?x=KX3_7QG]. One participant was excluded from the final sample since they completed the study on MTurk but were not compensated due to an error with their approval code.

Note that we purposefully overrecruited for adolescent and adult samples due to concerns related to unmoderated data collection.

### Procedure

**FIGURE 1:**
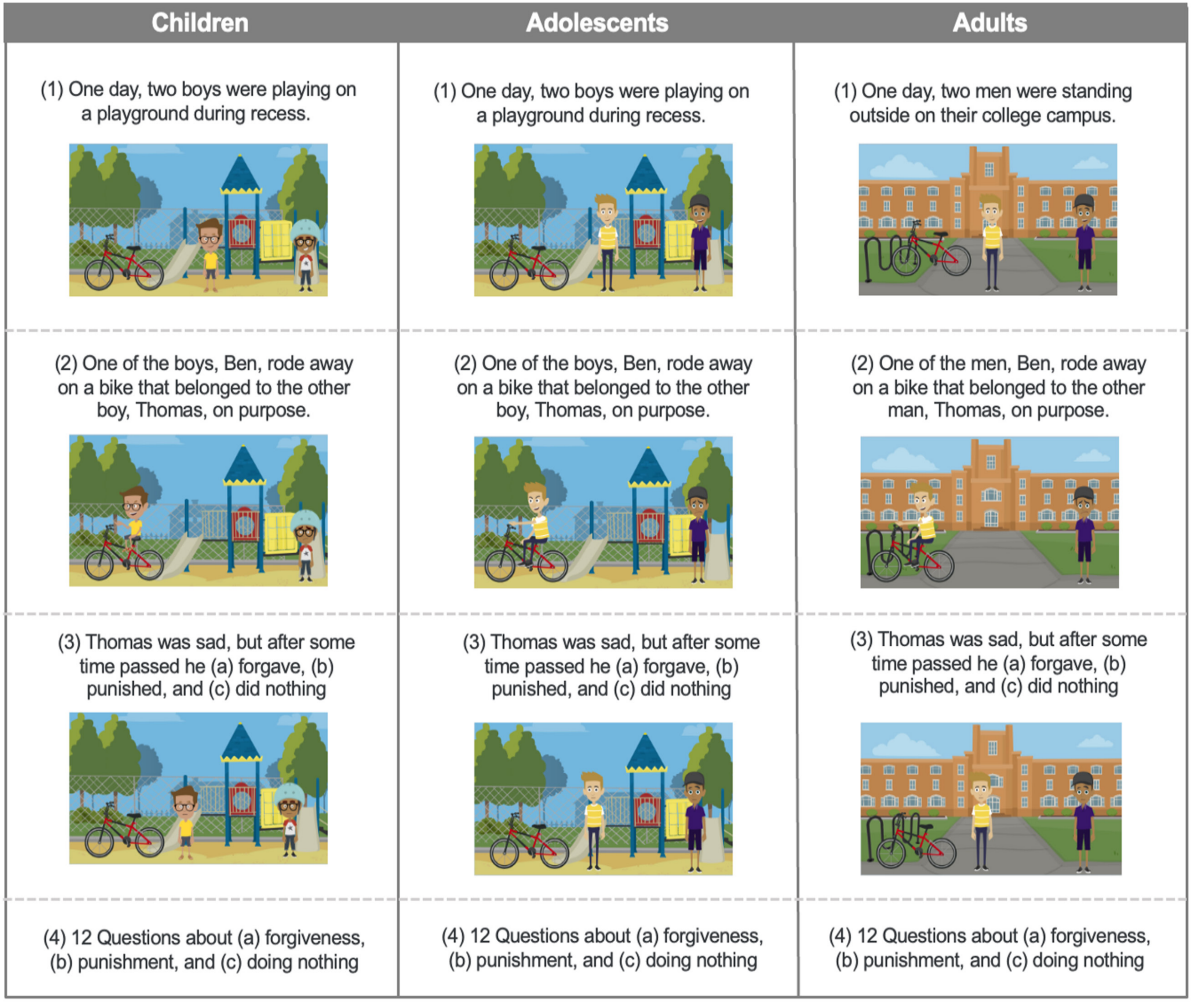
Study stimuli. Visual depiction of stimuli for one vignette for children, adolescents, and adults. © 2019 GoAnimate, Inc. Images are copyrighted by and used by permission of VYOND™. VYOND is a trademark of GoAnimate, Inc., registered in Australia, Brazil, the European Union, Norway, the Philippines, Singapore, Switzerland and the United Kingdom.

For child participants, an experimenter read three illustrated stories, in randomized order, to children over Zoom. Adolescent and adult participants, because they were tested in unmoderated study sessions, read all materials themselves. Each of the three stories depicted interpersonal transgressions between two individuals, who were shown and described as being the same age as the participants. All participants were shown stories where one character destroyed another character's clay sculpture (clay story), one character ripped another character's drawing (drawing story), and one character stole another character's bike (bike story; see Figure 1 for the images accompanying the bike story for our three age groups). Importantly, the response strategy was randomly paired with one of the three stories in order to control for any potential effects of the vignette. These stories depicted both male and female characters with varied skin tones and were pre‐tested with a sample of adult participants on Amazon Mechanical Turk to ensure similarity on the dimensions of frequency, severity, and morality.

Participants were first introduced to two characters (Figure 1, row 1) and were then told about a transgression committed by one of the characters (Figure 1, row 2). Specifically, in the bike story, participants were told: “One day, two boys were playing on the playground during recess. One of the boys, Ben, rode away on a bike that belonged to the other boy, Thomas, on purpose.” In the clay story, participants were told: “One day, two boys were making clay sculptures together during class. One of the boys, Jacob, took a clay sculpture from the other boy, David, and smashed it on purpose.” In the drawing story, participants were told: “One day, two girls were drawing pictures together during class. One of the girls, Emma, took a drawing from the other girl, Olivia, and ripped it in half on purpose.” Each story was accompanied by an image of a boy riding away on a bike (bike story), a boy knocking over a clay sculpture (clay story), or a girl crumpling up a drawing (drawing story). Participants saw vignettes depicting same‐age peers: children saw images of children, adolescents saw images of adolescents, and adults saw images of adults. Adult vignettes were described as occurring on a college campus rather than at a school.

After being introduced to the characters and the transgression in each story, participants were informed of the victim's response to the transgression (Figure 1, row 3). According to the Condition, participants were told that the victim forgave the offender (Forgiveness), punished the offender (Punishment), or decided not to do anything (Doing Nothing). In the Forgiveness Condition, participants were told: “[The victim] was sad, but after some time passed, [he/she] decided to forgive [the offender] for [transgression].” In the Punishment Condition, participants were told: “[The victim] was sad, but after some time passed, [he/she] decided to punish [the offender] for [transgression].” In the Doing Nothing Condition, participants were told: “[The victim] was sad, but after some time passed, [he/she] decided not to do anything.”

Participants first reported whether or not they believed a particular consequence (items listed in Table 1) would occur. Specifically, in the Forgiveness Condition, we asked, “Because [the victim] forgave [the offender], do you think [*consequence*]? Yes or no?”; in the Punishment Condition, we asked, “Because [the victim] punished [the offender], do you think [*consequence*]? Yes or no?”; and in the Doing Nothing Condition, we asked, “Do you think [*consequence*]? Yes or No?” We then asked how sure they were that the consequence would or would not occur (a tiny bit sure, kind of sure, or very sure). Each participant saw all 12 consequence items for each vignette, and the order of these was randomized for each participant. All participants saw three vignettes, each depicting a different response to the transgression. At the end of each testing session, participants also answered a memory check question (a free‐response question asking them to describe the victim's response in one of the three vignettes); however, in line with registered exclusion criteria, participants were not excluded for failing this memory check.

### Coding and analysis

For child participants, data were coded live in Qualtrics during experimental sessions by experimenters. For adolescent and adult participants, data were entered directly into Qualtrics by the participants themselves. For child participants, 26 experimental sessions (roughly 20%) were re‐coded. In this sample of trials (1838 trials in which both coders entered a value), there was strong agreement between live and video coding (*r*(1836) = .986, *p* < .001). When discrepancies were examined, they were largely due to errors on the part of the video coder rather than the live coder. Given the strong reliability, all analyses are based on the live‐coded values. Analyses were run in R version 3.6.3 (R Core Team, 2020). For all analyses, we used general linear models with endorsement as a dependent variable (−2.5 = “no, very sure,” −1.5 = “no, kind of sure,” −0.5 = “no, a little bit sure,” 0.5 = “yes, a little bit sure,” 1.5 = “yes, kind of sure,” 2.5 = “yes, very sure”).

For each item within each category (Table 1), we took a three‐fold approach to address our main research questions. This threefold approach corresponds with the three guiding research questions outlined in the introduction. To answer our first research question regarding whether participants distinguish between Conditions, we conducted an analysis of variance (ANOVA) with Condition (Forgiveness, Do Nothing, Punishment; within‐subjects) collapsing across Age Group (children, adolescents, and adults; between‐subjects). To answer our second research question regarding how individuals distinguish between response strategies, we examined pairwise comparisons between the three conditions for consequence items, where we found a main effect of Condition. We addressed our third research question regarding age‐related changes by conducting a 3 (Condition: Forgiveness, Doing Nothing, and Punishment) × 3 (Age Group: Children, Adolescents, and Adults) ANOVA for each item. If we found a significant interaction between Condition and Age Group, we looked for a simple effect of Age Group within each Condition and examined associated pairwise comparisons. We used Bonferroni corrections in R for pairwise comparisons; specifically, the *α* value for each comparison was divided by the number of comparisons being made in each test to correct for multiple comparisons. Estimates and *p*‐values for pairwise comparisons are included in Supporting Infomation (Tables S13–S25).

Although this analysis plan largely mirrors our pre‐registrations, it does deviate in one key way. In particular, we initially planned to examine the main effect of Condition on children, adolescents, and adults separately. Upon reflection, we instead opted to first collapse across these age groups to provide a picture of all participants' responses before focusing on Age Group differences. Nonetheless, we provide full analyses within each Age Group in the Supporting Infomation (Tables S1–S12; Figures S1–S4), in line with the pre‐registrations, and, importantly, those results cohere with the analyses presented here. We also conducted analyses looking at the interaction between Condition and Vignette, collapsed across Age Group, which are included in the Supporting Infomation (Table S26; Figures S5–S16).

In the subsequent description of results, we will use our four conceptual categories to organize analyses of the 12 consequence items. We opted for this approach rather than a factor analysis approach because we expected that how the items would cluster together would vary depending on Condition (i.e., forgiveness, punishment, and doing nothing) and thus it would not make sense to average across a subset of items without considering Condition. For each item in a category, we address our three research questions before contextualizing overall patterns.

In addition to the dependent measures described above, we also collected free responses from all participants where we asked them to describe what “punish” and “forgive” mean (“Can you tell me what you think the word ‘punish’ means?” and “Can you tell me what you think the word ‘forgive’ means?”). To explore participants' responses to these questions, we generated a frequency list based on the processing of their transcribed responses (children) or self‐entered responses (adolescents and adults). We initially attempted to code responses into conceptual categories, but reliability was so low that we opted instead to provide a bottom‐up description of the responses themselves (see Tables S27 and S28).

## RESULTS

### Positive behaviors

**FIGURE 2:**
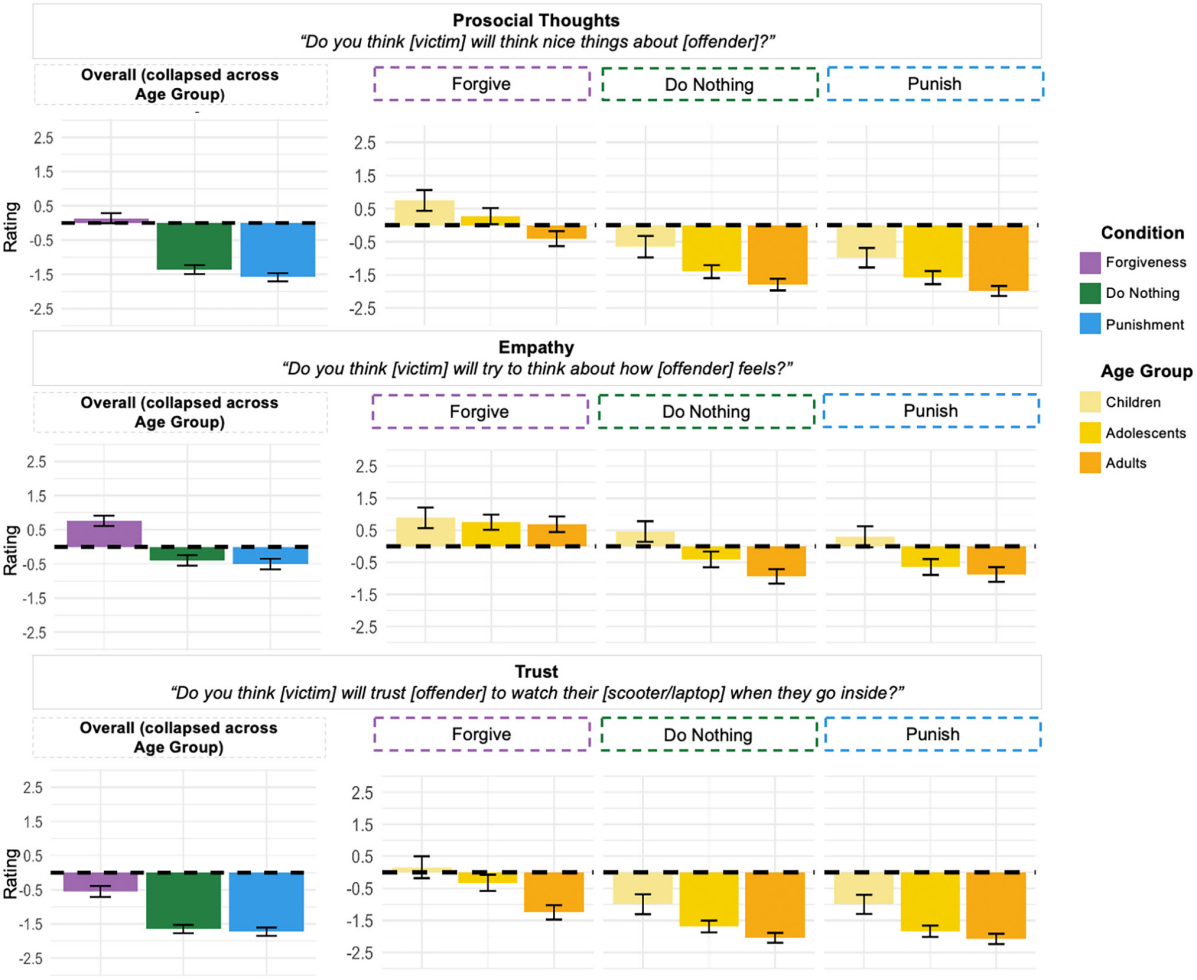
Effect of Condition and Age Group for positive behaviors. Participants' ratings as a function of Condition (forgiveness, do nothing, punishment) (on the left) and as a function of Condition (forgiveness, do nothing, punishment) (on the right) and Age Group (children, adolescents, adults) for the three items included in the “Positive Behaviors” category. *Y*‐axis shows likelihood judgments, with positive numbers representing affirmative answers and negative numbers representing negative answers. Error bars represent ±confidence intervals.

We first looked at results for Victim's Prosocial Thoughts about Offender, Victim's Empathy for Offender, and Victim's Future Trust of Offender (Figure 2).

For Victim's Prosocial Thoughts about the Offender, to answer our first research question regarding participants' differentiation between forgiveness, punishment, and doing nothing, we looked for the main effect of Condition. We found an effect of Condition (*F*(2, 1613) = 186.40, *p* < .001), indicating that our participants distinguished between the likelihood of prosocial thoughts after different interventions. To address our second research question regarding how participants differentiated between conditions, we looked at pairwise comparisons between the three conditions. Participants were more likely to predict prosocial thoughts about the offender after forgiveness (*M* = 0.14; SD = 0.07) compared to punishment (*M* = −1.58; SD = 0.07) and doing nothing (*M* = −1.36; SD = 0.07; *p*s < .001). To answer our third research question regarding the extent to which ratings differed by Age Group, we tested for a Condition × Age Group interaction and did not find an interaction effect (*F*(4, 1607) = 0.72, *p* = .578). Children, adolescents, and adults showed similar patterns in their ratings of prosocial thoughts about the offender.

For Victim's Empathy for the Offender, we also found an effect of Condition (*F*(2, 1612) = 81.01, *p* < .001). Participants were more likely to judge that a victim would feel empathy for the offender after forgiveness (*M* = 0.76; SD = 0.08) relative to punishment (*M* = −0.51; SD = 0.08) and doing nothing (*M* = −0.39; SD = 0.08; *p*s < .001). We found a Condition × Age Group interaction (*F*(4, 1606) = 5.41, *p* < .001), driven by an effect of Age Group for punishment (*F*(2, 537) = 18.14, *p* < .001) and doing nothing (*F*(2, 536) = 24.99, *p* < .001). Children were more likely than adolescents or adults to expect empathy for the offender after punishment or doing nothing, and adolescents were more likely than adults to expect empathy after the victim did nothing.

For Victim's Future Trust of the Offender, we found a significant effect of Condition (*F*(2, 1616) = 91.29, *p* < .001). Pairwise comparisons revealed that participants were more likely to expect a victim to trust the offender after forgiveness (*M* = −0.55; SD = 0.07) compared to punishment (*M* = −1.72; SD = 0.07) and doing nothing (*M* = −1.65; SD = 0.07; *p*s < .001). We found an interaction between Condition and Age Group (*F*(4, 1610) = 2.96, *p* = .019) and found simple effects of age for forgiveness (*F*(2, 538) = 26.42, *p* < .001), punishment (*F*(2, 537) = 25.66, *p* < .001), and doing nothing (*F*(2, 535) = 21.77, *p* < .001). Children were more likely than adolescents or adults to expect trust after all three interventions, and adolescents were more likely than adults to expect trust after the victim forgave or did nothing.

Summarizing the patterns of results we observed within the category of Positive Behaviors, we found that for all participants, forgiveness, punishment, and doing nothing were associated with different patterns of consequences. Forgiveness generated the highest ratings, and punishment and doing nothing generated lower ratings. We found interaction effects, with respect to Age, for Victim's Empathy for the Offender and Victim's Future Trust of the Offender, with children being more likely to expect these positive outcomes than adolescents or adults were after a victim was punished or did nothing.

### Negative behaviors

**FIGURE 3:**
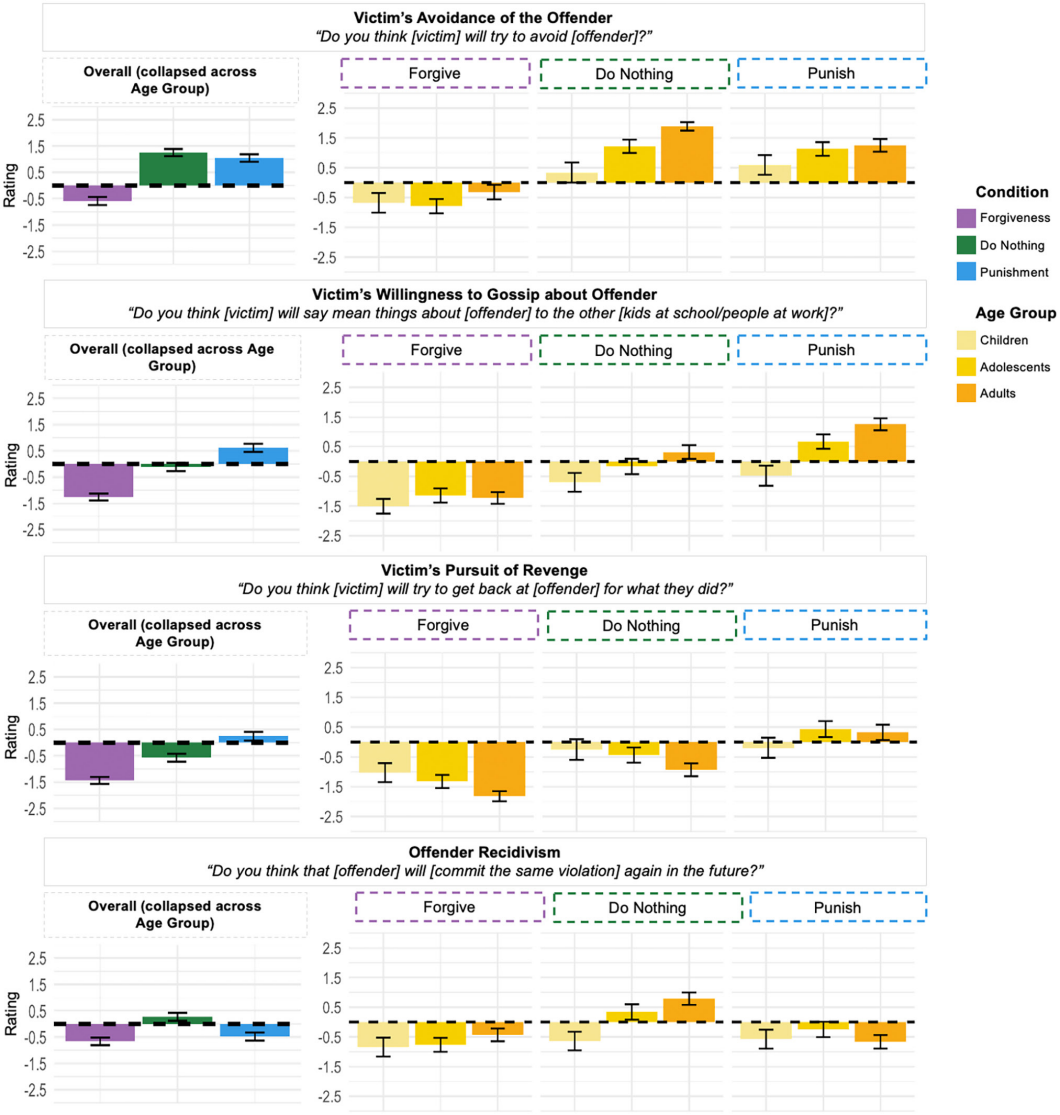
Effect of Condition and Age Group for negative behaviors. Participants' ratings as a function of Condition (forgiveness, do nothing, punishment) (on the left) and as a function of Condition (forgiveness, do nothing, punishment) (on the right) and Age Group (children, adolescents, adults) for the four items included in the “Negative Behaviors” category. *Y*‐axis shows likelihood judgments, with positive numbers representing affirmative answers and negative numbers representing negative answers. Error bars represent ±confidence intervals.

We next looked at results for Victim's Avoidance of the Offender, Victim's Willingness to Gossip about Offender, Victim's Pursuit of Revenge, and Offender Recidivism (Figure 3).

For Victim's Avoidance of the Offender, we found a significant effect of Condition (*F*(2, 1613) = 185.80, *p* < .001). The subsequent pairwise comparisons yielded significant results for the comparison between forgiveness (*M* = −0.59, SD = 0.07) and punishment (*M* = 1.05, SD = 0.07) and between forgiveness and doing nothing (*M* = 1.25, SD = 0.07; *p*s < .001). We found a significant interaction between Condition and Age Group (*F*(4, 1607) = 6.57, *p* < .001) and found simple effects of age for forgiveness (*F*(2, 534) = 3.75, *p* = .024), punishment (*F*(2, 536) = 6.40, *p* = .002), and doing nothing (*F*(2, 537) = 39.48, *p* < .001). After the victim forgave or did nothing, adolescents viewed avoidance as less likely than adults did, while after the victim punished or did nothing, children viewed avoidance as less likely than adolescents or adults did.

For Victim's Willingness to Gossip about the Offender, we found a significant main effect of Condition (*F*(2, 1615) = 157.00, *p* < .001). The victim's willingness to gossip was rated lowest after forgiveness (*M* = −1.26; SD = 0.08) and highest after punishment (*M* = 0.61, SD = 0.08), with intermediary ratings after doing nothing (*M* = −0.12, SD = 0.08; *p*s < .001). We found an interaction between Condition and Age Group (*F*(4, 1609) = 7.62, *p* < .001), and our subsequent analyses revealed a simple effect of Age Group for ratings of punishment (*F*(2, 537) = 40.07, *p* < .001) and doing nothing (*F*(2, 537) = 12.66, *p* < .001). After the victim was punished or did nothing, children were least likely to expect the victim to gossip, followed by adolescents and then adults.

For Victim's Pursuit of Revenge, we found an effect of Condition (*F*(2, 1610) = 120.20, *p* < .001). We found significant results for all pairwise comparisons between conditions (*p*s < .001). The victim's pursuit of revenge was rated least likely after forgiveness (*M* = −1.44, SD = 0.08) and most likely after punishment (*M* = 0.24, SD = 0.08), with intermediary ratings resulting after the victim did nothing (*M* = −0.57, SD = 0.08). We found an interaction between Condition and Age Group (*F*(4, 1604) = 6.76, *p* < .001), and found simple effects of age for forgiveness (*F*(2, 535) = 11.28, *p* < .001), punishment (*F*(2, 535) = 4.55, *p* = .011), and doing nothing (*F*(2, 534) = 6.66, *p* = .001). After forgiveness and doing nothing, children and adolescents viewed the victim's pursuit of revenge as less likely than adults did, while after punishment, children viewed revenge as less likely than adolescents did.

For Offender Recidivism, we found an effect of Condition (*F*(2, 1616) = 41.69, *p* < .001). Ratings were higher after doing nothing (*M* = 0.27, SD = 0.08) than after forgiveness (*M* = −0.66, SD = 0.08) and punishment (*M* = −0.48, SD = 0.08), suggesting that participants viewed forgiveness and punishment as similarly effective in dissuading future transgressions. We found an interaction between Condition and Age Group (*F*(4, 1610) = 9.32, *p* < .001) and found a simple effect of age for doing nothing (*F*(2, 535) = 26.82, *p* < .001), but not for forgiveness or punishment. After doing nothing, children were least likely to expect offender recidivism, followed by adolescents, and then adults.

Summarizing the overall pattern of results for negative behaviors, participants of all ages clearly differentiated between forgiveness, punishment, and doing nothing. Participants tended to rate negative outcomes as most likely following punishment and least likely following forgiveness. For most items in this category, ratings in the aftermath of doing nothing fell between those for forgiveness and punishment. However, Offender Recidivism was an exception: participants across our age groups viewed offender recidivism as more likely after the victim did nothing compared to after the victim forgave or was punished. This finding suggests that participants viewed both forgiveness and punishment as more effective in deterring future offenses than doing nothing. Age effects were found for all four Antisocial Behavior items, which were primarily driven by children's tendency to view these negative outcomes as less likely than adolescents or adults after a victim did nothing.

### Affective change

**FIGURE 4:**
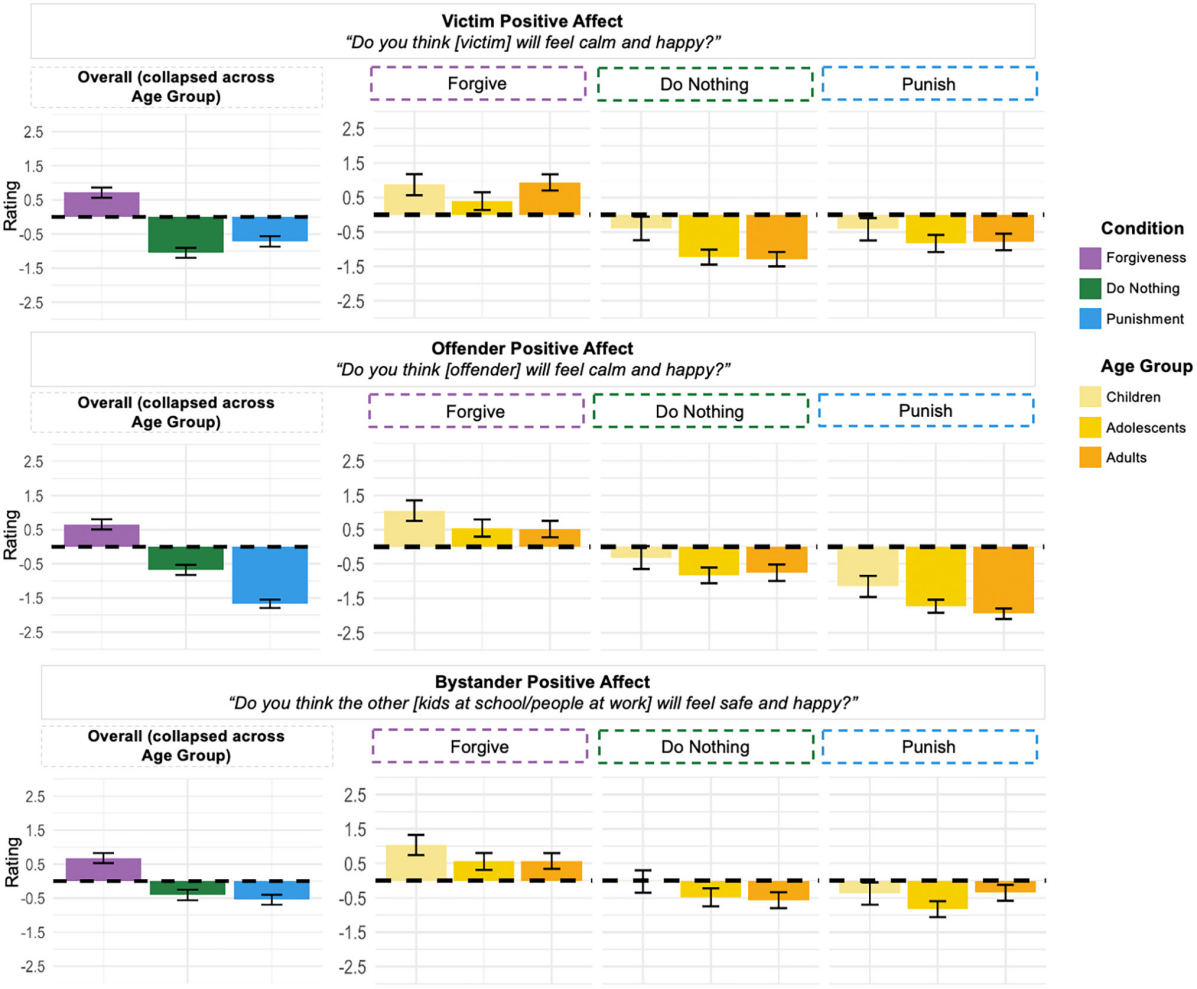
Effect of condition and age group for affective change. Participants' ratings as a function of Condition (forgiveness, do nothing, punishment) (on the left) and as a function of Condition (forgiveness, do nothing, punishment) (on the right) and Age Group (children, adolescents, adults) for the three items included in the “Affective Change” category. *Y*‐axis shows likelihood judgments, with positive numbers representing affirmative answers and negative numbers representing negative answers. Error bars represent ±confidence intervals.

Next, we looked at the pattern of results for Affective Change, including Victim Positive Affect, Offender Positive Affect, and Bystander Positive Affect (Figure 4).

For Victim Positive Affect, we found an effect of Condition (*F*(2, 1609) = 150.80, *p* < .001), indicating that our participants distinguished between these interventions. We found effects for all pairwise comparisons (*p*s < .010). Participants rated the likelihood of the victim feeling positive emotions highest when victims forgave (*M* = 0.71, SD = 0.08) and lowest when victims did nothing (*M* = −1.05, SD = 0.08), with the rating for punishment falling between these two (*M* = −0.72, SD = 0.08). Our test for an interaction between Age Group and Condition was significant (*F*(4, 1603) = 3.52, *p* = .007), and subsequent tests for effects of age found effects for forgiveness (*F*(2, 535) = 5.43, *p* = .005) and doing nothing (*F*(2, 533) = 13.20, *p* < .001). After the victim forgave, adolescents viewed their positive affect as less likely than adults did, while after the victim did nothing, children viewed their positive affect as more likely than adolescents or adults did.

For Offender Positive Affect, we found an effect of Condition (*F*(2, 1610) = 266.00, *p* < .001). We found significant effects for all pairwise comparisons (*p*s < .001). Participants rated positive offender affect as most likely in the aftermath of forgiveness (*M* = 0.66, SD = 0.07) and least likely in the aftermath of punishment (*M* = −1.67, SD = 0.07), with doing nothing (*M* = −0.68, SD = 0.07) falling between. We did not find an interaction between Condition and Age Group (*F*(4, 1604) = 0.64, *p* = .635).

For Bystander Positive Affect, we found an effect of Condition (*F*(2, 1612) = 77.82, *p* < .001). Bystander positive affect was rated more likely after forgiveness (*M* = 0.68, SD = 0.08) than after punishment (*M* = −0.55, SD = 0.08) or doing nothing (*M* = −0.41, SD = 0.08; *p*s < .001). We did not find an interaction between Condition and Age Group (*F*(4, 1606) = 1.86, *p* = .114).

Summarizing the overall patterns of results for Affective Change, we found that participants perceived forgiveness to be most effective in promoting positive affective changes for involved parties and viewed punishment and doing nothing as relatively ineffective. We found an interaction between age and intervention type only for victim positive affect, suggesting that, in general, participants made similar assessments, regardless of age, about the likelihood of offender and bystander positive affect.

### Others' interest in affiliation

**FIGURE 5:**
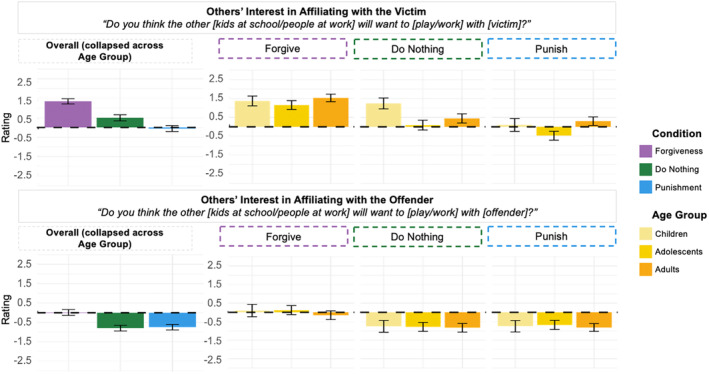
Effect of Condition and Age Group for others' interest in affiliation. Participants' ratings as a function of Condition (forgiveness, do nothing, punishment) (on the left) and as a function of Condition (forgiveness, do nothing, punishment) (on the right) and Age Group (children, adolescents, adults) for the two items included in the “Others' Interest in Affiliation” category. *Y*‐axis shows likelihood judgments, with positive numbers representing affirmative answers and negative numbers representing negative answers. Error bars represent ±confidence intervals.

Last, we looked at results for Others' Interest in Affiliating with the Victim and Others' Interest in Affiliating with the Offender (Figure 5).

For Others' Interest in Affiliating with the Victim, we found an effect of Condition (*F*(2, 1610) = 88.52, *p* < .001). Participants reported that others would be most likely to affiliate with victims after forgiveness (*M* = 1.34, SD = 0.08) and least likely to affiliate with victims after punishment (*M* = −0.06, SD = 0.07), with intermediary results after doing nothing (*M* = 0.49, SD = 0.07; *p*s < .001). We found an interaction between Condition and Age Group (*F*(4, 1604) = 5.20, *p* < .001). In testing for simple effects of Age Group, we found effects for punishment (*F*(2, 536) = 10.40, *p* < .001) and doing nothing (*F*(2, 536) = 16.52, *p* < .001). After punishment, children and adults were more likely than adolescents to expect others to affiliate with the victim, while after doing nothing, children were more likely to expect affiliation with the victim than were adults and adolescents.

For Others' Interest in Affiliating with the Offender, we found an effect of Condition (*F*(2, 1618) = 36.44, *p* < .001). Ratings were highest in the aftermath of forgiveness (*M* = 0.01, SD = 0.07) and lower in the aftermath of both punishment (*M* = −0.74, SD = 0.07) and doing nothing (*M* = −0.79, SD = 0.07; *p*s < .001). We did not find an interaction between Condition and Age Group (*F*(4, 1612) = 0.28, *p* = .892).

To summarize the overall pattern of results for Others' Interest in Affiliating with the Victim, we found that ratings were highest for forgiveness and lowest for punishment, meaning that participants thought that others were most likely to affiliate with victims and offenders after forgiveness and least likely to affiliate with victims and offenders after punishment. We found interaction effects for Interest in Affiliating with the Victim, primarily driven by children's higher ratings, relative to adolescents and adults, after punishment or doing nothing.

### Free responses

With respect to definitions of punishment, both adolescents and adults tended to define punishment as a consequence of wrongdoing or as an act of revenge, with terms like “consequence,” “penalty,” “revenge,” and “retribution” being used frequently. Children tended to define punishment by referring to its antisocial nature and to the nature of the transgression, with terms like “bad,” “mean,” and “trouble” being used frequently. With respect to definitions of forgiveness, all age groups tended to reference apologies and acceptance of apologies, with terms like “accept,” “okay,” “sorry,” and “apology” being used frequently. Adolescents and adults also tended to define forgiveness as moving on, letting go, or forgetting about a misdeed, with terms like “let [go],” “move,” “past,” and “forget” being used frequently (see Tables S27 and S28 for more details). These data can be interpreted in conjunction with previous work from Enright (1994), which sought to explore children's, adolescents', and adults' beliefs about the conditions that must precede forgiveness. In particular, young children's and adolescents' use of the terms “sorry” and “apologize” may suggest that younger participants in our sample viewed these conditions as necessary for forgiveness to occur. Moreover, our results bear some resemblance to data from Wainryb et al. (2019), who asked 7‐, 11‐, and 16‐year‐olds to define forgiveness and found that 7‐year‐olds were more likely than older children to refer to social consequences, such as relationship repair, while older children were more likely to reference psychological changes.

### Memory check

As described above, all participants were asked a memory check question at the end of the task. In particular, they were asked to describe the victim's response in one of the stories (clay sculpture). Despite being a free‐response recall question, our memory check was answered correctly by the majority of participants. 163 adult participants (out of 198), 135 adolescent participants (out of 217), and 60 child participants (out of 129) answered the memory check correctly. Although rates are lower among child participants versus adolescent and adult participants, the fact that all child participants were tested in a moderated session allows us to be more confident that failing this check was more related to deficits in working memory than to attentional focus.

## GENERAL DISCUSSION

In the present study, we addressed three key research questions. First, do individuals distinguish between the consequences of forgiveness, punishment, and doing nothing? Second, where do we observe differences in the consequences associated with these response strategies? And third, for which of these consequences do we observe age‐related changes, and what is the nature of these changes?

### Key findings

We found that participants from our three age samples displayed significant differences in the consequences associated with forgiveness, punishment, and doing nothing. We found an effect of Condition for all 12 of the items included in our study, providing robust evidence that, across a broad age range, people tend to view the consequences of forgiveness, punishment, and doing nothing as distinct from one another.

To unpack the nature of these distinctions, we first observed general patterns in the directionality of participant ratings in each of the four categories we introduced to scaffold our hypotheses. First, for Positive Behaviors, we found that participants rated the likelihood of these consequences higher after forgiveness than after punishment or doing nothing. Second, for negative behaviors, participants rated negative consequences as least likely after forgiveness and relatively more likely after punishment and doing nothing. Third, for Affective Change, we observed that positive affect in victims, offenders, and bystanders was rated most likely after forgiveness and least likely after punishment. Fourth and finally, for Others' Interest in Affiliating, participants rated the likelihood of others' interest in affiliating with victims and offenders highest after forgiveness and lower after punishment and doing nothing. Together, these results suggest participants systematically distinguished between the behavioral and affective consequences of forgiveness, punishment, and doing nothing.

To address our second question, which asked how individuals distinguished between these response strategies, we observed relatively consistent patterns in our pairwise comparisons between forgiveness, punishment, and doing nothing for the 12 items. For nearly all the items included in the study, forgiveness led to significantly different ratings than punishment or doing nothing. Forgiveness was perceived as the most likely to lead to positive behavioral consequences and the least likely to lead to negative behavioral consequences. In contrast, ratings following punishment were not significantly different from ratings following doing nothing for half of the items. When compared to forgiveness, punishment and doing nothing were both perceived as less likely to promote positive consequences and more likely to promote negative consequences. This pattern of results suggests that, far from viewing forgiveness as simply refraining from punishment, participants perceived forgiveness as having unique positive consequences relative to the two other response strategies.

To address our third research question, we tested three different age samples using the same methodology, allowing us to examine age‐related differences in perceptions of the consequences associated with forgiveness, punishment, and doing nothing. We observed interaction effects for eight of the 12 items we included in this study: Victim's Trust, Victim's Empathy for the Offender, Victim's Avoidance, Victim's Willingness to Gossip, Victim's Pursuit of Revenge, Offender Recidivism, Victim Positive Affect, and Others' Interest in Affiliating with the Victim. For most items where we found age‐related effects as a function of the response strategy, this variation was a result of differences between children's ratings, compared to adults' and adolescents', for punishment and doing nothing but not for forgiveness. Age differences were found for forgiveness in only five of the items for which we observed an interaction between Condition and Age, suggesting that, in general, perceptions of forgiveness remain relatively stable throughout development. We also found that even young children differentiated between the three response strategies. This finding provides evidence that experience with transgressions and associated responses may increase individuals' tendency to differentiate between various responses, although the ability to do so is present even in the youngest participants.

Importantly, although our child participants tended to, in general, view forgiveness as leading to positive consequences and punishment and doing nothing as leading to negative consequences, our data does point to some more nuanced distinctions. In particular, even our child participants tended to view Offender Recidivism as more likely after the victim did nothing compared to after the victim punished or forgave the offender. Thus, children in our study were able to recognize that punishment has value in deterring future offenses, although it also is expected to generate negative consequences in other domains.

Our results connect to existing literature by providing additional insights into factors underlying children's, adolescents', and adults' evaluations of and engagement in forgiveness and punishment. In line with previous research pointing to children's and adults' positive attitudes toward forgiving victims and preferences for forgiving over unforgiving victims (Oostenbroek & Vaish, 2019a, 2019b), our participants tended to view positive outcomes as most likely in the aftermath of forgiveness. The finding that participants across our three age groups tended to view negative outcomes as more likely in the aftermath of punishment also aligns with some previous work showing that children negatively evaluate victims who engage in second‐party punishment and prefer alternative response strategies to punishment when making third‐party decisions (e.g., Strauß & Bondü, 2022; Yang et al., 2021). The results of the present study generate several important theoretical questions. We will address some of these remaining questions here, as well as share our thoughts regarding potential answers.

First, why do our participants perceive the consequences of punishment and doing nothing as so similar? While our initial study design was motivated in part by the goal of determining if participants distinguished between the outcomes of forgiveness and doing nothing, we in fact found that punishment and doing nothing were most similar. There are several distinct explanations for why we may have observed these patterns of results. One of these is participants' interpretation of a victim's decision to do nothing and their attribution of negatively valenced emotions and motivations in the aftermath of a transgression. Our participants may have assumed that both the punishing victim and the victim who did nothing were similarly upset by the offense and motivated to inflict harm, but that the victim who did nothing was able to restrain their behavior. If the difference between these two victims was perceived predominantly as a difference in behavior, not an internal state, this may explain why punishment and doing nothing yielded such similar patterns of ratings.

Second, why do we see more developmental change in punishment and doing nothing compared to forgiveness? The data from the present study cannot shed light on the mechanisms underlying this observation, but based on existing literature, we can speculate on some potential explanations. One possible reason for this finding is that younger children in our sample may have displayed a general positivity bias in their expectations following interpersonal transgressions (Boseovski, 2010; Lockhart et al., 2009). In general, children may have assumed that victims would harbor fewer negative emotions, regardless of the response strategy. This positivity bias may have resulted in children having more positive expectations about individuals' interactions across conditions, but since these other age groups (i.e., adolescents, adults) also viewed forgiveness as generating positive outcomes, age differences were less prominent for forgiveness. In this way, the present findings suggest that social learning may result in children recognizing the negative outcomes associated with punishment and doing nothing while simultaneously maintaining children's initial inclinations that forgiveness is associated with positive outcomes. In support of this possibility, children also tend to view punishment as a path to redemption (Dunlea & Heiphetz, 2021), which may lead children, more than adolescents or adults, to infer that any response to a transgression would promote positive and obstruct negative consequences.

Another potential explanation for the developmental differences we observed is the cognitive and psychological changes that are occurring over the course of the lifespan. Between the age ranges in our child, adolescent, and adult samples, there are important psychological developments that may have influenced the way participants interpreted the consequences of forgiving, punishing, and doing nothing. In the Introduction, we have identified several cognitive factors that we view as potentially relevant in the ways our participants conceive of punishment, forgiveness, and doing nothing (e.g., theory of mind, cognitive flexibility, and emotional comprehension), many of which would develop or strengthen over the lifespan.

Finally, the free‐response data we generated by asking participants to define the words “punish” and “forgive” generates valuable insights into how children, adolescents, and adults conceptualize these response strategies. For instance, with respect to definitions of forgiveness, all age groups tended to reference apologies, while adolescents and adults also used terms evoking concepts of letting go and moving on. These trends indicate a shift toward a more nuanced concept of forgiveness and a change from a focus on required conditions for forgiveness to the emotional components of forgiveness, in line with Enright's process model of forgiveness (Enright, 1994; Enright et al., 1989; Enright & Song, 2020). While not the focus of the current project, these findings may be informative in future studies investigating the development of intervention‐related attitudes and behaviors and provide initial insights into how participants operationalize forgiveness and punishment throughout the lifespan.

### Limitations

It is important to recognize some limitations of the current research. First, we must acknowledge that the set of consequence items included in the design, while based on measures used in existing literature (Table 1), did not result from a pre‐established theoretical framework or a factor analysis. Indeed, a factor analysis approach was not theoretically possible in the current design, as we predicted that the items would cluster in different ways depending on the response strategy, so additional research would be required to better ascertain how children and adults think about the items independent of the intervention. Nonetheless, we included a vast array of diverse consequence items with the goal of capturing a broad range of outcomes. Although there are limitations to casting such a wide net, we were still able to document differences between the interventions and developmental changes for the items we included.

Second, the current study only assessed the perceived consequences of forgiveness, punishment, and doing nothing. Of course, there exist myriad other intervention strategies in the aftermath of interpersonal transgressions; in particular, helping has been shown to be the preferred form of intervention in several developmental studies (e.g., Lee & Warneken, 2020). Future studies can compare the perceived consequences of forgiveness, punishment, and doing nothing to those of compensation, partner choice, and protesting. These studies can include more hypothesis‐driven sets of dependent variables in order to gain insight into how consequences beyond those measured here may vary with response strategy. Moreover, future research should explore the interrelationship between various response strategies and, in particular, the relationship between interventions such as punishment, compensation, and forgiveness. Some research with adult participants (e.g., Wenzel & Okimoto, 2014) has suggested that punishment can be conducive to forgiveness by promoting perceptions of justice for victims, but no research, to our knowledge, has tested this relationship with child participants. Future work in this area will generate a more comprehensive understanding of children's expectations following diverse responses to transgressions, as well as shed light on why children may choose to engage in one strategy over others. It will also be valuable to build on the results of our free‐response questions by using more direct questioning to test a priori hypotheses regarding how individuals understand forgiveness and punishment across a broad age range.

Third, although the current study tested participants from three different age groups, there are still limitations in the generalizability of our findings. Despite our best efforts to recruit a diverse sample of participants, we limited our samples to the United States, which prevents us from reaching broader conclusions about how individuals in other cultures perceive the consequences of these interventions. There were also likely unmeasured demographic differences between our three samples, such as geographical location, race and ethnicity, religiosity, and political orientation. Future work can fill these gaps by conducting research outside of the United States, and in particular in societies where punishment may be more or less valued or frequently enacted, as well as in more varied communities and cultures within the United States. Incorporating cross‐cultural approaches may enable us to understand how various experiential and socialization factors influence these expectations.

Fourth, due to the nature of our design, we cannot be fully confident that participants did not use descriptions of the victims' interventions to infer more general information about a victim's characteristics and behavior. For example, the subjects in our study may have perceived “forgivers” as categorically different from “punishers” (or “nothing‐doers”) and associated different behaviors with these types of victims. Participants in our study indicated that forgiveness is more likely to be associated with positive emotions and behaviors relative to punishment or doing nothing, but the present data does not allow us to evaluate the causal relationship here. Future research can incorporate questions about participants' impressions of victims in relation to their behavior and thus better elucidate how victim characteristics or behaviors are linked to particular consequences.

Finally, we acknowledge that our design could have better integrated attentional and comprehension checks. Although we included a memory check at the end of the session, this check did not directly assess attention, and a more direct assessment could help establish the extent to which our participants, in particular adolescents and adults (who completed the study asynchronously), were attuned to conditional differences.

## CONCLUSION

In summary, the present study examined perceptions of the consequences associated with three interventions for transgressions. Our results indicated that, regardless of age, participants distinguished between the consequences associated with forgiveness, punishment, and doing nothing and did so in meaningful ways. Forgiveness was most likely to be associated with positive behavioral and affective consequences, while punishment and forgiveness were more likely to be associated with negative consequences. These findings generate important questions regarding why punishment and doing nothing are perceived as so similar across the range of items in this study, as well as why forgiveness is so distinct. We also observed age‐related differences, with children providing different ratings than adolescents and adults in the aftermath of punishment and doing nothing. Children provided higher ratings than older participants for positively‐valenced items and lower ratings for negatively‐valenced items in these two conditions, which leads to questions about the mechanisms underlying these developmental shifts. The results of this study help us better understand what individuals expect to occur in the aftermath of forgiveness and punishment and how these expectations change over development.

## CONFLICT OF INTEREST STATEMENT

We have no known conflict of interest to disclose.

## Supporting information


Appendix S1.

## Data Availability

The data, analytic code, and materials necessary to reproduce the analyses presented in this paper are publicly accessible, and the analyses presented here are preregistered. The data, code, materials, and preregistration links are available at the following URL: https://osf.io/hmd4z/?view_only=2f8d13ed3cb7451caac37930288c9137.
