# Supplementary material for: Developing conceptions of forgiveness across the lifespan
Source: Child Dev. 2024 May 31;95(6):1915–33. doi: 10.1111/cdev.14122 (PMC11579636; doi:10.1111/cdev.14122)

**Supporting information for *Developing conceptions of forgiveness across the lifespan***

**Table of Content**

[**Table S1.** Statistics for simple effect of Condition within each Age Group and pairwise comparisons for Victim Prosocial Behavior. 4](#_Toc155609015)

[**Table S2.** Statistics for simple effect of Condition within each Age Group and pairwise comparisons for Victim’s Empathy for the Offender. 5](#_Toc155609016)

[**Table S3.** Statistics for simple effect of Condition within each Age Group and pairwise comparisons for Victim’s Future Trust of the Offender. 6](#_Toc155609017)

[**Table S4.** Statistics for simple effect of Condition within each Age Group and pairwise comparisons for Victim’s Avoidance of the Offender. 7](#_Toc155609018)

[**Table S5.** Statistics for simple effect of Condition within each Age Group and pairwise comparisons for Victim’s Willingness to Gossip about the Offender. 8](#_Toc155609019)

[**Table S6.** Statistics for simple effect of Condition within each Age Group and pairwise comparisons for Victim’s Pursuit of Revenge. 9](#_Toc155609020)

[**Table S7.** Statistics for simple effect of Condition within each Age Group and pairwise comparisons for Offender Recidivism. 10](#_Toc155609021)

[**Table S8.** Statistics for simple effect of Condition within each Age Group and pairwise comparisons for Victim Affective Change. 11](#_Toc155609022)

[**Table S9.** Statistics for simple effect of Condition within each Age Group and pairwise comparisons for Offender Affective Change. 12](#_Toc155609023)

[**Table S10.** Statistics for simple effect of Condition within each Age Group and pairwise comparisons for Bystander Affective Change. 13](#_Toc155609024)

[**Table S11.** Statistics for simple effect of Condition within each Age Group and pairwise comparisons for Others’ Willingness to Interact with the Victim. 14](#_Toc155609025)

[**Table S12.** Statistics for simple effect of Condition within each Age Group and pairwise comparisons for Others’ Willingness to Interact with the Offender. 15](#_Toc155609026)

[**Table S13.** Statistics for simple effect of Age Group within each Condition and pairwise comparisons for Victim Prosocial Behavior. 16](#_Toc155609027)

[**Table S14.** Statistics for simple effect of Age Group within each Condition and pairwise comparisons for Victim Empathy for the Offender. 17](#_Toc155609028)

[**Table S15.** Statistics for simple effect of Age Group within each Condition and pairwise comparisons for Victim’s Future Trust of the Offender. 18](#_Toc155609029)

[**Table S16.** Statistics for simple effect of Age Group within each Condition and pairwise comparisons for Victim’s Avoidance of the Offender. 19](#_Toc155609030)

[**Table S17.** Statistics for simple effect of Age Group within each Condition and pairwise comparisons for Victim’s Willingness to Gossip about the Offender. 20](#_Toc155609031)

[**Table S18.** Statistics for simple effect of Age Group within each Condition and pairwise comparisons for Victim’s Pursuit of Revenge. 21](#_Toc155609032)

[**Table S19.** Statistics for simple effect of Age Group within each Condition and pairwise comparisons for Offender Recidivism. 22](#_Toc155609033)

[**Table S20.** Statistics for simple effect of Age Group within each Condition and pairwise comparisons for Victim Affective Change. 23](#_Toc155609034)

[**Table S21.** Statistics for simple effect of Age Group within each Condition and pairwise comparisons for Offender Affective Change. 24](#_Toc155609035)

[**Table S22.** Statistics for simple effect of Age Group within each Condition and pairwise comparisons for Bystander Affective Change. 25](#_Toc155609036)

[**Table S23.** Statistics for simple effect of Age Group within each Condition and pairwise comparisons for Others’ Willingness to Interact with the Victim. 26](#_Toc155609037)

[**Table S24.** Statistics for simple effect of Age Group within each Condition and pairwise comparisons for Others’ Willingness to Interact with the Offender. 27](#_Toc155609038)

[**Table 25** | Statistical values for main effects, interaction effects, and simple effects for each item. 28](#_Toc155609039)

[**Table 26** | Statistical values for main effects of Vignette and interaction effects between Vignette and Condition for each item. 29](#_Toc155609040)

[**Table S27.** Word frequency for children’s, adolescents’, and adults’ free-response answers to the question, “Can you tell me what you think the word ‘punish’ means?”. 30](#_Toc155609041)

[**Table S28.** Word frequency for children’s, adolescents’, and adults’ free-response answers to the question, “Can you tell me what you think the word ‘forgive’ means?”. 32](#_Toc155609042)

[**Figure S1.** Participants’ ratings as a function of Condition (punishment, do nothing, forgiveness) (on the left) and as a function of Age Group (children, adolescents, adults) and Condition (punishment, do nothing, forgiveness) (on the right) for the three items included in the “Prosocial Thoughts and Emotions” category. 34](#_Toc155609043)

[**Figure S2.** Participants’ ratings as a function of Condition (punishment, do nothing, forgiveness) (on the left) and as a function of Age Group (children, adolescents, adults) and Condition (punishment, do nothing, forgiveness) (on the right) for the three items included in the “Antisocial Behaviors” category. 35](#_Toc155609044)

[**Figure S3.** Participants’ ratings as a function of Condition (punishment, do nothing, forgiveness) (on the left) and as a function of Age Group (children, adolescents, adults) and Condition (punishment, do nothing, forgiveness) (on the right) for the three items included in the “Affective Change” category. 36](#_Toc155609045)

[**Figure S4.** Participants’ ratings as a function of Condition (punishment, do nothing, forgiveness) (on the left) and as a function of Age Group (children, adolescents, adults) and Condition (punishment, do nothing, forgiveness) (on the right) for the three items included in the “Affiliative Interest” category. Error bars represent +/- confidence intervals. 37](#_Toc155609046)

[**Figure S5.** Participants’ ratings as a function of Condition (punishment, do nothing, forgiveness) and Vignette (Bike, Clay, Drawing) for Victim Prosocial Behavior. 38](#_Toc155609047)

[**Figure S6.** Participants’ ratings as a function of Condition (punishment, do nothing, forgiveness) and Vignette (Bike, Clay, Drawing) for Victim’s Empathy for the Offender. 39](#_Toc155609048)

[**Figure S7.** Participants’ ratings as a function of Condition (punishment, do nothing, forgiveness) and Vignette (Bike, Clay, Drawing) for Victim’s Trust of the Offender. 40](#_Toc155609049)

[**Figure S8.** Participants’ ratings as a function of Condition (punishment, do nothing, forgiveness) and Vignette (Bike, Clay, Drawing) for Victim’s Avoidance of the Offender. 41](#_Toc155609050)

[**Figure S9.** Participants’ ratings as a function of Condition (punishment, do nothing, forgiveness) and Vignette (Bike, Clay, Drawing) for Victim’s Willingness to Gossip About the Offender. 42](#_Toc155609051)

[**Figure S10.** Participants’ ratings as a function of Condition (punishment, do nothing, forgiveness) and Vignette (Bike, Clay, Drawing) for Victim’s Pursuit of Revenge. 43](#_Toc155609052)

[**Figure S11.** Participants’ ratings as a function of Condition (punishment, do nothing, forgiveness) and Vignette (Bike, Clay, Drawing) for Offender Recidivism. 44](#_Toc155609053)

[**Figure S12.** Participants’ ratings as a function of Condition (punishment, do nothing, forgiveness) and Vignette (Bike, Clay, Drawing) for Victim Affective Change. 45](#_Toc155609054)

[**Figure S13.** Participants’ ratings as a function of Condition (punishment, do nothing, forgiveness) and Vignette (Bike, Clay, Drawing) for Offender Affective Change. 46](#_Toc155609055)

[**Figure S14.** Participants’ ratings as a function of Condition (punishment, do nothing, forgiveness) and Vignette (Bike, Clay, Drawing) for Bystander Affective Change. 47](#_Toc155609056)

[**Figure S15.** Participants’ ratings as a function of Condition (punishment, do nothing, forgiveness) and Vignette (Bike, Clay, Drawing) for Others’ Evaluation of the Victim. 48](#_Toc155609057)

[**Figure S16.** Participants’ ratings as a function of Condition (punishment, do nothing, forgiveness) and Vignette (Bike, Clay, Drawing) for Others’ Evaluation of the Offender. 49](#_Toc155609058)

# **Table S1.** Statistics for simple effect of Condition within each Age Group and pairwise comparisons for Victim Prosocial Behavior.

Prosocial Behavior

| Age Group | *F*-value | *p*-value | Contrasts | | |
| --- | --- | --- | --- | --- | --- |
|  |  |  |  | Estimate | *p*-value |
| Children | *F*(2, 384)=34.25 | *p* < .001* | Forgive – Do Nothing | -1.40 | <.001* |
|  |  |  | Forgive – Punish | -1.73 | <.001* |
|  |  |  | Do Nothing – Punish | -0.33 | .400 |
|  |  |  |  |  |  |
| Adolescents | *F*(2, 634)=90.83 | *p* < .001* | Forgive – Do Nothing | -1.68 | <.001* |
|  |  |  | Forgive – Punish | -1.85 | <.001* |
|  |  |  | Do Nothing – Punish | -0.18 | .716 |
|  |  |  |  |  |  |
| Adults | *F*(2, 589)=83.06 | *p* < .001* | Forgive – Do Nothing | -1.39 | <.001* |
|  |  |  | Forgive – Punish | -1.58 | <.001* |
|  |  |  | Do Nothing – Punish | -1.93 | .450 |

# **Table S2.** Statistics for simple effect of Condition within each Age Group and pairwise comparisons for Victim’s Empathy for the Offender.

Empathy

| Age Group | *F*-value | *p*-value | Contrasts | | |
| --- | --- | --- | --- | --- | --- |
|  |  |  |  | Estimate | *p*-value |
| Children | *F*(2, 384)=3.46 | *p*=.032* | Forgive – Do Nothing | -0.43 | .198 |
|  |  |  | Forgive – Punish | -0.59 | .034* |
|  |  |  | Do Nothing – Punish | -0.16 | 1.00 |
|  |  |  |  |  |  |
| Adolescents | *F*(2, 634)=36.46 | *p* < .001* | Forgive – Do Nothing | -1.16 | <.001* |
|  |  |  | Forgive – Punish | -1.40 | <.001* |
|  |  |  | Do Nothing – Punish | -0.23 | .540 |
|  |  |  |  |  |  |
| Adults | *F*(2, 588)=65.55 | *p* < .001* | Forgive – Do Nothing | -1.62 | <.001* |
|  |  |  | Forgive – Punish | -1.57 | <.001* |
|  |  |  | Do Nothing – Punish | 0.06 | 1.00 |

# **Table S3.** Statistics for simple effect of Condition within each Age Group and pairwise comparisons for Victim’s Future Trust of the Offender.

Trust

| Age Group | *F*-value | *p*-value | Contrasts | | |
| --- | --- | --- | --- | --- | --- |
|  |  |  |  | Estimate | *p*-value |
| Children | *F*(2, 384)=17.16 | *p* < .001* | Forgive – Do Nothing | -1.16 | <.001* |
|  |  |  | Forgive – Punish | -1.16 | <.001* |
|  |  |  | Do Nothing – Punish | < 0.01 | 1.00 |
|  |  |  |  |  |  |
| Adolescents | *F*(2, 637)=61.92 | *p* < .001* | Forgive – Do Nothing | -1.36 | <.001* |
|  |  |  | Forgive – Punish | -1.51 | <.001* |
|  |  |  | Do Nothing – Punish | -0.15 | .949 |
|  |  |  |  |  |  |
| Adults | *F*(2, 589)=25.75 | *p* < .001* | Forgive – Do Nothing | -0.79 | <.001* |
|  |  |  | Forgive – Punish | -0.83 | <.001* |
|  |  |  | Do Nothing – Punish | -0.03 | 1.00 |

# **Table S4.** Statistics for simple effect of Condition within each Age Group and pairwise comparisons for Victim’s Avoidance of the Offender.

Avoidance

| Age Group | *F*-value | *p*-value | Contrasts | | |
| --- | --- | --- | --- | --- | --- |
|  |  |  |  | Estimate | *p*-value |
| Children | *F*(2, 384)=15.98 | *p* < .001* | Forgive – Do Nothing | 1.02 | <.001* |
|  |  |  | Forgive – Punish | 1.27 | <.001* |
|  |  |  | Do Nothing – Punish | 0.26 | .849 |
|  |  |  |  |  |  |
| Adolescents | *F*(2, 633)=93.08 | *p* < .001* | Forgive – Do Nothing | 2.01 | <.001* |
|  |  |  | Forgive – Punish | 1.92 | <.001* |
|  |  |  | Do Nothing – Punish | -0.09 | 1.00 |
|  |  |  |  |  |  |
| Adults | *F*(2, 590)=119.6 | *p* < .001* | Forgive – Do Nothing | 2.20 | <.001* |
|  |  |  | Forgive – Punish | 1.57 | <.001* |
|  |  |  | Do Nothing – Punish | -0.63 | <.001* |

# **Table S5.** Statistics for simple effect of Condition within each Age Group and pairwise comparisons for Victim’s Willingness to Gossip about the Offender.

Willingness to Gossip

| Age Group | *F*-value | *p*-value | Contrasts | | |
| --- | --- | --- | --- | --- | --- |
|  |  |  |  | Estimate | *p*-value |
| Children | *F*(2, 384)=12.46 | *p* < .001* | Forgive – Do Nothing | 0.81 | <.001* |
|  |  |  | Forgive – Punish | 1.03 | <.001* |
|  |  |  | Do Nothing – Punish | 0.23 | .904 |
|  |  |  |  |  |  |
| Adolescents | *F*(2, 635)=52.73 | *p* < .001* | Forgive – Do Nothing | 0.98 | <.001* |
|  |  |  | Forgive – Punish | 1.82 | <.001* |
|  |  |  | Do Nothing – Punish | 0.84 | <.001* |
|  |  |  |  |  |  |
| Adults | *F*(2, 590)=138.0 | *p* < .001* | Forgive – Do Nothing | 1.55 | <.001* |
|  |  |  | Forgive – Punish | 2.48 | <.001* |
|  |  |  | Do Nothing – Punish | 0.94 | <.001* |

# **Table S6.** Statistics for simple effect of Condition within each Age Group and pairwise comparisons for Victim’s Pursuit of Revenge.

Pursuit of Revenge

| Age Group | *F*-value | *p*-value | Contrasts | | |
| --- | --- | --- | --- | --- | --- |
|  |  |  |  | Estimate | *p*-value |
| Children | *F*(2, 383)=7.58 | *p* < .001* | Forgive – Do Nothing | 0.78 | .004* |
|  |  |  | Forgive – Punish | 0.83 | .002* |
|  |  |  | Do Nothing – Punish | 0.06 | 1.00 |
|  |  |  |  |  |  |
| Adolescents | *F*(2, 633)=48.90 | *p* < .001* | Forgive – Do Nothing | 0.89 | <.001* |
|  |  |  | Forgive – Punish | 1.76 | <.001* |
|  |  |  | Do Nothing – Punish | 0.87 | <.001* |
|  |  |  |  |  |  |
| Adults | *F*(2, 588)=96.55 | *p* < .001* | Forgive – Do Nothing | 0.89 | <.001* |
|  |  |  | Forgive – Punish | 2.14 | <.001* |
|  |  |  | Do Nothing – Punish | 1.26 | <.001* |

# **Table S7.** Statistics for simple effect of Condition within each Age Group and pairwise comparisons for Offender Recidivism.

Offender Recidivism

| Age Group | *F*-value | *p*-value | Contrasts | | |
| --- | --- | --- | --- | --- | --- |
|  |  |  |  | Estimate | *p*-value |
| Children | *F*(2, 384)=0.45 | *p* = .74 | Forgive – Do Nothing | 0.20 | 1.00 |
|  |  |  | Forgive – Punish | 0.26 | 1.00 |
|  |  |  | Do Nothing – Punish | 0.06 | 1.00 |
|  |  |  |  |  |  |
| Adolescents | *F*(2, 637)=18.75 | *p* < .001* | Forgive – Do Nothing | 1.11 | <.001* |
|  |  |  | Forgive – Punish | 0.52 | .012* |
|  |  |  | Do Nothing – Punish | -0.59 | .004* |
|  |  |  |  |  |  |
| Adults | *F*(2, 592)=49.18 | *p* < .001* | Forgive – Do Nothing | 1.22 | <.001* |
|  |  |  | Forgive – Punish | -0.22 | .496 |
|  |  |  | Do Nothing – Punish | -1.43 | <.001* |

# **Table S8.** Statistics for simple effect of Condition within each Age Group and pairwise comparisons for Victim Affective Change.

Victim Affective Change

| Age Group | *F*-value | *p*-value | Contrasts | | |
| --- | --- | --- | --- | --- | --- |
|  |  |  |  | Estimate | *p*-value |
| Children | *F*(2, 384)=20.39 | *p* < .001* | Forgive – Do Nothing | -1.27 | <.001* |
|  |  |  | Forgive – Punish | -1.29 | <.001* |
|  |  |  | Do Nothing – Punish | -0.02 | 1.00 |
|  |  |  |  |  |  |
| Adolescents | *F*(2, 631)=47.71 | *p* < .001* | Forgive – Do Nothing | -1.63 | <.001* |
|  |  |  | Forgive – Punish | -1.23 | <.001* |
|  |  |  | Do Nothing – Punish | 0.40 | .069 |
|  |  |  |  |  |  |
| Adults | *F*(2, 588)=101.5 | *p* < .001* | Forgive – Do Nothing | -2.23 | <.001* |
|  |  |  | Forgive – Punish | -1.73 | <.001* |
|  |  |  | Do Nothing – Punish | 0.50 | .007* |

# **Table S9.** Statistics for simple effect of Condition within each Age Group and pairwise comparisons for Offender Affective Change.

Offender Affective Change

| Age Group | *F*-value | *p*-value | Contrasts | | |
| --- | --- | --- | --- | --- | --- |
|  |  |  |  | Estimate | *p*-value |
| Children | *F*(2, 384)=49.30 | *p* < .001* | Forgive – Do Nothing | -1.36 | <.001* |
|  |  |  | Forgive – Punish | -2.20 | <.001* |
|  |  |  | Do Nothing – Punish | -0.84 | <.001* |
|  |  |  |  |  |  |
| Adolescents | *F*(2, 630)  =101.60 | *p* < .001* | Forgive – Do Nothing | -1.38 | <.001* |
|  |  |  | Forgive – Punish | -2.27 | <.001* |
|  |  |  | Do Nothing – Punish | -0.89 | <.001* |
|  |  |  |  |  |  |
| Adults | *F*(2, 590)  =128.40 | *p* < .001* | Forgive – Do Nothing | -1.27 | <.001* |
|  |  |  | Forgive – Punish | -2.46 | <.001* |
|  |  |  | Do Nothing – Punish | -1.19 | <.001* |

# **Table S10.** Statistics for simple effect of Condition within each Age Group and pairwise comparisons for Bystander Affective Change.

Bystander Affective Change

| Age Group | *F*-value | *p*-value | Contrasts | | |
| --- | --- | --- | --- | --- | --- |
|  |  |  |  | Estimate | *p*-value |
| Children | *F*(2, 384)=21.43 | *p* < .001* | Forgive – Do Nothing | -1.06 | <.001* |
|  |  |  | Forgive – Punish | -1.41 | <.001* |
|  |  |  | Do Nothing – Punish | -0.35 | .363 |
|  |  |  |  |  |  |
| Adolescents | *F*(2, 632)=33.43 | *p* < .001* | Forgive – Do Nothing | -1.04 | <.001* |
|  |  |  | Forgive – Punish | -1.39 | <.001* |
|  |  |  | Do Nothing – Punish | -0.34 | .157 |
|  |  |  |  |  |  |
| Adults | *F*(2, 590)=26.94 | *p* < .001* | Forgive – Do Nothing | -1.14 | <.001* |
|  |  |  | Forgive – Punish | -0.93 | <.001* |
|  |  |  | Do Nothing – Punish | 0.22 | .565 |

# **Table S11.** Statistics for simple effect of Condition within each Age Group and pairwise comparisons for Others’ Willingness to Interact with the Victim.

Others’ Willingness to Interact with Victim

| Age Group | *F*-value | *p*-value | Contrasts | | |
| --- | --- | --- | --- | --- | --- |
|  |  |  |  | Estimate | *p*-value |
| Children | *F*(2, 384)=21.45 | *p* < .001* | Forgive – Do Nothing | -0.13 | 1.00 |
|  |  |  | Forgive – Punish | -1.27 | <.001* |
|  |  |  | Do Nothing – Punish | -1.14 | <.001* |
|  |  |  |  |  |  |
| Adolescents | *F*(2, 634)=44.41 | *p* < .001* | Forgive – Do Nothing | -1.06 | <.001* |
|  |  |  | Forgive – Punish | -1.62 | <.001* |
|  |  |  | Do Nothing – Punish | -0.56 | .004* |
|  |  |  |  |  |  |
| Adults | *F*(2, 586)=35.19 | *p* < .001* | Forgive – Do Nothing | -1.09 | <.001* |
|  |  |  | Forgive – Punish | -1.24 | <.001* |
|  |  |  | Do Nothing – Punish | -0.15 | 1.00 |

# **Table S12.** Statistics for simple effect of Condition within each Age Group and pairwise comparisons for Others’ Willingness to Interact with the Offender.

Others’ Willingness to Interact with Offender

| Age Group | *F*-value | *p*-value | Contrasts | | |
| --- | --- | --- | --- | --- | --- |
|  |  |  |  | Estimate | *p*-value |
| Children | *F*(2, 384)=9.17 | *p* < .001* | Forgive – Do Nothing | -0.85 | <.001* |
|  |  |  | Forgive – Punish | -0.84 | <.001* |
|  |  |  | Do Nothing – Punish | 0.01 | 1.00 |
|  |  |  |  |  |  |
| Adolescents | *F*(2, 639)=16.03 | *p* < .001* | Forgive – Do Nothing | -0.91 | <.001* |
|  |  |  | Forgive – Punish | -0.80 | <.001* |
|  |  |  | Do Nothing – Punish | 0.11 | 1.00 |
|  |  |  |  |  |  |
| Adults | *F*(2, 589)=11.26 | *p* < .001* | Forgive – Do Nothing | -0.68 | <.001* |
|  |  |  | Forgive – Punish | -0.66 | <.001* |
|  |  |  | Do Nothing – Punish | 0.02 | 1.00 |

# **Table S13.** Statistics for simple effect of Age Group within each Condition and pairwise comparisons for Victim Prosocial Behavior.

Prosocial Behavior

| Condition | *F*-value | *p*-value | Contrasts | | |
| --- | --- | --- | --- | --- | --- |
|  |  |  |  | Estimate | *p*-value |
| Forgive | *F*(2, 537)=18.46 | *p* < .001* | Children – Adolescents | -0.47 | .043* |
|  |  |  | Children – Adults | -1.15 | <.001* |
|  |  |  | Adolescents – Adults | -0.68 | <.001* |
|  |  |  |  |  |  |
| Do Nothing | *F*(2, 534)=23.25 | *p* < .001* | Children – Adolescents | -0.75 | <.001* |
|  |  |  | Children – Adults | -1.14 | <.001* |
|  |  |  | Adolescents – Adults | -0.39 | .025* |
|  |  |  |  |  |  |
| Punish | *F*(2, 536)=20.29 | *p* < .001* | Children – Adolescents | -0.60 | <.001* |
|  |  |  | Children – Adults | -1.00 | <.001* |
|  |  |  | Adolescents – Adults | -0.40 | .011* |

# **Table S14.** Statistics for simple effect of Age Group within each Condition and pairwise comparisons for Victim Empathy for the Offender.

Empathy

| Condition | *F*-value | *p*-value | Contrasts | | |
| --- | --- | --- | --- | --- | --- |
|  |  |  |  | Estimate | *p*-value |
| Forgive | *F*(2, 533)=0.51 | *p* = .602 | Children – Adolescents | -0.14 | 1.00 |
|  |  |  | Children – Adults | -0.20 | .953 |
|  |  |  | Adolescents – Adults | -0.05 | 1.00 |
|  |  |  |  |  |  |
| Do Nothing | *F*(2, 536)=24.99 | *p* < .001* | Children – Adolescents | -0.87 | <.001* |
|  |  |  | Children – Adults | -1.40 | <.001* |
|  |  |  | Adolescents – Adults | -0.53 | .007* |
|  |  |  |  |  |  |
| Punish | *F*(2, 537)=18.14 | *p* < .001* | Children – Adolescents | -0.94 | <.001* |
|  |  |  | Children – Adults | -1.18 | <.001* |
|  |  |  | Adolescents – Adults | -0.24 | .542 |

# **Table S15.** Statistics for simple effect of Age Group within each Condition and pairwise comparisons for Victim’s Future Trust of the Offender.

Trust

| Condition | *F*-value | *p*-value | Contrasts | | |
| --- | --- | --- | --- | --- | --- |
|  |  |  |  | Estimate | *p*-value |
| Forgive | *F*(2, 538)=26.42 | *p* < .001* | Children – Adolescents | -0.49 | .048* |
|  |  |  | Children – Adults | -1.41 | <.001* |
|  |  |  | Adolescents – Adults | -0.92 | <.001* |
|  |  |  |  |  |  |
| Do Nothing | *F*(2, 535)=21.77 | *p* < .001* | Children – Adolescents | -0.69 | <.001* |
|  |  |  | Children – Adults | -1.05 | <.001* |
|  |  |  | Adolescents – Adults | -0.35 | .034* |
|  |  |  |  |  |  |
| Punish | *F*(2, 537)=25.66 | *p* < .001* | Children – Adolescents | -0.84 | <.001* |
|  |  |  | Children – Adults | -1.08 | <.001* |
|  |  |  | Adolescents – Adults | -0.24 | .235 |

# **Table S16.** Statistics for simple effect of Age Group within each Condition and pairwise comparisons for Victim’s Avoidance of the Offender.

Avoidance

| Condition | *F*-value | *p*-value | Contrasts | | |
| --- | --- | --- | --- | --- | --- |
|  |  |  |  | Estimate | *p*-value |
| Forgive | *F*(2, 534)=3.75 | *p* = .024* | Children – Adolescents | -0.11 | 1.00 |
|  |  |  | Children – Adults | 0.36 | .229 |
|  |  |  | Adolescents – Adults | 0.47 | .024* |
|  |  |  |  |  |  |
| Do Nothing | *F*(2, 537)=39.48 | *p* < .001* | Children – Adolescents | 0.88 | <.001* |
|  |  |  | Children – Adults | 1.55 | <.001* |
|  |  |  | Adolescents – Adults | 0.67 | <.001* |
|  |  |  |  |  |  |
| Punish | *F*(2, 536)=6.40 | *p* = .002* | Children – Adolescents | 0.50 | .023* |
|  |  |  | Children – Adults | 0.66 | .002* |
|  |  |  | Adolescents – Adults | 0.16 | 1.00 |

# **Table S17.** Statistics for simple effect of Age Group within each Condition and pairwise comparisons for Victim’s Willingness to Gossip about the Offender.

Willingness to Gossip

| Condition | *F*-value | *p*-value | Contrasts | | |
| --- | --- | --- | --- | --- | --- |
|  |  |  |  | Estimate | *p*-value |
| Forgive | *F*(2, 535)=2.26 | *p* = .105 | Children – Adolescents | 0.36 | .111 |
|  |  |  | Children – Adults | 0.28 | .335 |
|  |  |  | Adolescents – Adults | -0.08 | 1.00 |
|  |  |  |  |  |  |
| Do Nothing | *F*(2, 537)=12.66 | *p* < .001* | Children – Adolescents | 0.51 | .035* |
|  |  |  | Children – Adults | 1.02 | <.001* |
|  |  |  | Adolescents – Adults | 0.51 | .011* |
|  |  |  |  |  |  |
| Punish | *F*(2, 537)=40.07 | *p* < .001* | Children – Adolescents | 1.15 | <.001* |
|  |  |  | Children – Adults | 1.73 | <.001* |
|  |  |  | Adolescents – Adults | 0.58 | .002* |

# **Table S18.** Statistics for simple effect of Age Group within each Condition and pairwise comparisons for Victim’s Pursuit of Revenge.

Pursuit of Revenge

| Condition | *F*-value | *p*-value | Contrasts | | |
| --- | --- | --- | --- | --- | --- |
|  |  |  |  | Estimate | *p*-value |
| Forgive | *F*(2, 535)=11.28 | *p* < .001* | Children – Adolescents | -0.30 | .247 |
|  |  |  | Children – Adults | -0.79 | <.001* |
|  |  |  | Adolescents – Adults | -0.49 | .004* |
|  |  |  |  |  |  |
| Do Nothing | *F*(2, 534)=6.66 | *p* = .001* | Children – Adolescents | -0.19 | 1.00 |
|  |  |  | Children – Adults | -0.68 | .003* |
|  |  |  | Adolescents – Adults | -0.50 | .016* |
|  |  |  |  |  |  |
| Punish | *F*(2, 535)=4.55 | *p* = .011* | Children – Adolescents | 0.63 | .011* |
|  |  |  | Children – Adults | 0.52 | .053 |
|  |  |  | Adolescents – Adults | -0.11 | 1.00 |

# **Table S19.** Statistics for simple effect of Age Group within each Condition and pairwise comparisons for Offender Recidivism.

Offender Recidivism

| Condition | *F*-value | *p*-value | Contrasts | | |
| --- | --- | --- | --- | --- | --- |
|  |  |  |  | Estimate | *p*-value |
| Forgive | *F*(2, 536)=2.92 | *p* = .055 | Children – Adolescents | 0.07 | 1.00 |
|  |  |  | Children – Adults | 0.41 | .102 |
|  |  |  | Adolescents – Adults | 0.33 | .140 |
|  |  |  |  |  |  |
| Do Nothing | *F*(2, 535)=26.82 | *p* < .001* | Children – Adolescents | 0.98 | <.001* |
|  |  |  | Children – Adults | 1.43 | <.001* |
|  |  |  | Adolescents – Adults | 0.45 | .027* |
|  |  |  |  |  |  |
| Punish | *F*(2, 539)=3.01 | *p* = .050 | Children – Adolescents | 0.33 | .309 |
|  |  |  | Children – Adults | -0.09 | 1.00 |
|  |  |  | Adolescents – Adults | -0.42 | .058 |

# **Table S20.** Statistics for simple effect of Age Group within each Condition and pairwise comparisons for Victim Affective Change.

Victim Affective Change

| Condition | *F*-value | *p*-value | Contrasts | | |
| --- | --- | --- | --- | --- | --- |
|  |  |  |  | Estimate | *p*-value |
| Forgive | *F*(2, 535)=5.43 | *p* = .005* | Children – Adolescents | -0.48 | .052 |
|  |  |  | Children – Adults | 0.07 | 1.00 |
|  |  |  | Adolescents – Adults | 0.54 | .007* |
|  |  |  |  |  |  |
| Do Nothing | *F*(2, 533)=13.20 | *p* < .001* | Children – Adolescents | -0.83 | <.001* |
|  |  |  | Children – Adults | -0.89 | <.001* |
|  |  |  | Adolescents – Adults | -0.06 | 1.00 |
|  |  |  |  |  |  |
| Punish | *F*(2, 535)=2.35 | *p* = .097 | Children – Adolescents | -0.41 | .121 |
|  |  |  | Children – Adults | -0.37 | .216 |
|  |  |  | Adolescents – Adults | 0.05 | 1.00 |

# **Table S21.** Statistics for simple effect of Age Group within each Condition and pairwise comparisons for Offender Affective Change.

Offender Affective Change

| Condition | *F*-value | *p*-value | Contrasts | | |
| --- | --- | --- | --- | --- | --- |
|  |  |  |  | Estimate | *p*-value |
| Forgive | *F*(2, 532)=4.34 | *p* = .014* | Children – Adolescents | -0.51 | .030* |
|  |  |  | Children – Adults | -0.54 | .022* |
|  |  |  | Adolescents – Adults | -0.03 | 1.00 |
|  |  |  |  |  |  |
| Do Nothing | *F*(2, 535)=3.79 | *p* = .023* | Children – Adolescents | -0.52 | .025* |
|  |  |  | Children – Adults | -0.44 | .082 |
|  |  |  | Adolescents – Adults | 0.08 | 1.00 |
|  |  |  |  |  |  |
| Punish | *F*(2, 537)=12.87 | *p* < .001* | Children – Adolescents | -0.58 | <.001* |
|  |  |  | Children – Adults | -0.79 | <.001* |
|  |  |  | Adolescents – Adults | -0.22 | .339 |

# **Table S22.** Statistics for simple effect of Age Group within each Condition and pairwise comparisons for Bystander Affective Change.

Bystander Affective Change

| Condition | *F*-value | *p*-value | Contrasts | | |
| --- | --- | --- | --- | --- | --- |
|  |  |  |  | Estimate | *p*-value |
| Forgive | *F*(2, 534)=3.72 | *p* = .025 | Children – Adolescents | -0.48 | .038 |
|  |  |  | Children – Adults | -0.46 | .051 |
|  |  |  | Adolescents – Adults | 0.01 | 1.00 |
|  |  |  |  |  |  |
| Do Nothing | *F*(2, 535)=3.84 | *p* = .022* | Children – Adolescents | -0.46 | .072 |
|  |  |  | Children – Adults | -0.54 | .025* |
|  |  |  | Adolescents – Adults | -0.08 | 1.00 |
|  |  |  |  |  |  |
| Punish | *F*(2, 537)=4.72 | *p* = .009* | Children – Adolescents | -0.45 | .057 |
|  |  |  | Children – Adults | 0.02 | 1.00 |
|  |  |  | Adolescents – Adults | 0.48 | .016* |

# **Table S23.** Statistics for simple effect of Age Group within each Condition and pairwise comparisons for Others’ Willingness to Interact with the Victim.

Others’ Willingness to Interact with Victim

| Condition | *F*-value | *p*-value | Contrasts | | |
| --- | --- | --- | --- | --- | --- |
|  |  |  |  | Estimate | *p*-value |
| Forgive | *F*(2, 532)=3.01 | *p* = .050 | Children – Adolescents | -0.22 | .648 |
|  |  |  | Children – Adults | 0.16 | 1.00 |
|  |  |  | Adolescents – Adults | 0.38 | .044* |
|  |  |  |  |  |  |
| Do Nothing | *F*(2, 536)=16.52 | *p* < .001* | Children – Adolescents | -1.15 | <.001* |
|  |  |  | Children – Adults | -0.79 | <.001* |
|  |  |  | Adolescents – Adults | 0.35 | .141 |
|  |  |  |  |  |  |
| Punish | *F*(2, 536)=10.49 | *p* < .001* | Children – Adolescents | -0.57 | .012* |
|  |  |  | Children – Adults | 0.20 | .956 |
|  |  |  | Adolescents – Adults | 0.77 | <.001* |

# **Table S24.** Statistics for simple effect of Age Group within each Condition and pairwise comparisons for Others’ Willingness to Interact with the Offender.

Others’ Willingness to Interact with the Offender

| Condition | *F*-value | *p*-value | Contrasts | | |
| --- | --- | --- | --- | --- | --- |
|  |  |  |  | Estimate | *p*-value |
| Forgive | *F*(2, 537)=1.41 | *p* = .245 | Children – Adolescents | 0.03 | 1.00 |
|  |  |  | Children – Adults | -0.25 | .640 |
|  |  |  | Adolescents – Adults | -0.28 | .352 |
|  |  |  |  |  |  |
| Do Nothing | *F*(2, 538)=0.08 | *p* = .927 | Children – Adolescents | -0.03 | 1.00 |
|  |  |  | Children – Adults | -0.07 | 1.00 |
|  |  |  | Adolescents – Adults | -0.05 | 1.00 |
|  |  |  |  |  |  |
| Punish | *F*(2, 537)=0.32 | *p* = .725 | Children – Adolescents | 0.07 | 1.00 |
|  |  |  | Children – Adults | -0.07 | 1.00 |
|  |  |  | Adolescents – Adults | -0.13 | 1.00 |

| **Table 25** \| Statistical values for main effects, interaction effects, and simple effects for each item. Significant *p*-values are indicated with “*”. | | | | | | | | | |
| --- | --- | --- | --- | --- | --- | --- | --- | --- | --- |
|  | Main Effect of Condition | Pairwise Comparison Differences | Age Group x Condition Interaction | Effect of Age Group within Condition | | | Effect of Condition within Age Group | | |
|  |  |  |  | Forgive | Do Nothing | Punish | Children | Adolescents | Adults |
|  |  |  |  |  |  |  |  |  |  |
| Prosocial Behavior | *F*(2,1613) =  186.40  *p* < .001* | Forgive > Punish = Do Nothing | *F*(4, 1607) = 0.72  *p* = .578 | *F*(2, 537) = 18.46  *p* <.001* | *F*(2, 534) = 23.25  *p* < .001* | *F*(2, 536) = 20.29  *p* < .001* | *F*(2, 384) = 34.25 *p* < .001* | *F*(2, 634) = 90.83 *p* < .001* | *F*(2, 589) = 83.06 *p* < .001* |
|  |  |  |  |  |  |  |  |  |  |
| Empathy | *F*(2,1612) =  81.01  *p* < .001* | Forgive > Punish = Do Nothing | *F*(4, 1606) = 5.41 *p* < .001* | *F*(2, 533) = 0.51  *p* = .602 | *F*(2, 536) = 24.99  *p* < .001* | *F*(2, 537) = 18.14  *p* < .001* | *F*(2, 384) = 3.46 *p* = .032* | *F*(2, 634) = 36.46 *p* < .001* | *F*(2, 588) = 61.30 *p* < .001* |
|  |  |  |  |  |  |  |  |  |  |
| Trust | *F*(2,1616) =  91.29  *p* < .001* | Forgive > Punish = Do Nothing | *F*(4, 1610) = 2.96  *p* = .019* | *F*(2, 538) = 26.42  *p* < .001* | *F*(2, 535) = 21.77  *p* < .001* | *F*(2, 537) = 25.66  *p* < .001* | *F*(2, 384) = 17.16 *p* < .001* | *F*(2, 637) = 61.92 *p* < .001* | *F*(2,589)=25.75 *p* < .001* |
|  |  |  |  |  |  |  |  |  |  |
| Avoidance | *F*(2,1613) =  185.80  *p* < .001* | Punish = Do Nothing > Forgive | *F*(4, 1607) = 6.57  *p* < .001* | *F*(2, 534) = 3.75  *p* = .024* | *F*(2, 537) = 39.48  *p* < .001* | *F*(2, 536) = 6.40  *p* = .002* | *F*(2, 384) = 15.98 *p* < .001* | *F*(2, 633) = 93.08 *p* < .001* | *F*(2, 590)=119.60 *p* < .001* |
|  |  |  |  |  |  |  |  |  |  |
| Willingness to Gossip | *F*(2,1615) =  157.00  *p* < .001* | Punish > Do Nothing > Forgive | *F*(4, 1609) = 7.62  *p* < .001* | *F*(2, 535) = 2.26  *p* = .105 | *F*(2, 537) = 12.66  *p* < .001* | *F*(2, 537) = 40.07  *p* < .001* | *F*(2, 384) = 12.46 *p* < .001* | *F*(2, 635) = 52.73 *p* < .001* | *F*(2, 590)=138.00 *p* < .001* |
|  |  |  |  |  |  |  |  |  |  |
| Pursuit of Revenge | *F*(2,1610) = 120.20  *p* < .001* | Punish > Do Nothing > Forgive | *F*(4, 1604) = 6.76  *p* < .001* | *F*(2, 535) = 11.28  *p* < .001* | *F*(2, 534) = 6.66  *p* = .001* | *F*(2, 535) = 4.55  *p* = .011* | *F*(2, 383) = 7.58 *p* < .001* | *F*(2, 654) = 48.90 *p* < .001* | *F*(2, 588)=95.00 *p* < .001* |
|  |  |  |  |  |  |  |  |  |  |
| Offender Recidivism | *F*(2,1616) =  41.69  *p* < .001* | Do Nothing > Punish = Forgive | *F*(4, 1610) = 9.32  *p* < .001* | *F*(2, 536) = 2.92  *p* = .055 | *F*(2, 535) = 26.83  *p* < .001* | *F*(2, 539) = 3.01  *p* = .050 | *F*(2, 384) = 0.74 *p* = .478 | *F*(2, 637) = 18.75 *p* < .001* | *F*(2, 589)=50.26 *p* < .001* |
|  |  |  |  |  |  |  |  |  |  |
| Victim Positive Affect | *F*(2,1609) =  150.80  *p* < .001* | Forgive > Punish > Do Nothing | *F*(4, 1603) = 3.52  *p* = .007* | *F*(2, 535) = 5.43  *p* = .004* | *F*(2, 533) = 13.20  *p* < .001* | *F*(2, 535) = 2.35  *p* = .097 | *F*(2, 384) = 20.39  *p* < .001* | *F*(2, 631) = 47.71  *p* < .001* | *F*(2, 588)=101.5 *p* < .001* |
|  |  |  |  |  |  |  |  |  |  |
| Offender Positive Affect | *F*(2,1610) =  266.00  *p* < .001* | Forgive > Do Nothing > Punish | *F*(4, 1604) = 0.64  *p* = .635 | *F*(2, 532) = 4.364  *p* = .014* | *F*(2, 535) = 3.79  *p* = .023* | *F*(2, 537) = 12.87  *p* < .001* | *F*(2, 384) = 49.30  *p* < .001* | *F*(2, 630) = 101.60  *p* < .001* | *F*(2, 590)=128.40 *p* < .001* |
|  |  |  |  |  |  |  |  |  |  |
| Bystander Positive Affect | *F*(2,1612) =  77.82  *p* < .001* | Forgive > Punish = Do Nothing | *F*(4, 1604) = 1.86  *p* = .110 | *F*(2, 534) = 3.72  *p* = .025* | *F*(2, 535) = 3.84  *p* = .022* | *F*(2, 537) = 4.72  *p* = .009* | *F*(2, 384) = 21.43  *p* < .001* | *F*(2, 632) = 33.43  *p* < .001* | *F*(2, 590)=26.94 *p* < .001* |
|  |  |  |  |  |  |  |  |  |  |
| Others’ Interest in Affiliating with Victim | *F*(2,1610) =  88.52  *p* < .001* | Forgive > Do Nothing > Punish | *F*(4, 1604) = 5.20  *p* < .001* | *F*(2, 532) = 3.01  *p* = .050 | *F*(2, 536) = 16.52  *p* < .001* | *F*(2, 536) = 10.40  *p* < .001* | *F*(2, 384) = 21.45  *p* < .001* | *F*(2, 634) = 44.41 *p* < .001* | *F*(2, 586)=35.19 *p* < .001* |
|  |  |  |  |  |  |  |  |  |  |
| Others’ Interest in Affiliating with Offender | *F*(2,1618) =  36.44  *p* < .001* | Forgive > Punish = Do Nothing | *F*(4, 1612) = 0.28  *p* = .892 | *F*(2, 537) = 1.41  *p* = .245 | *F*(2, 538) = 0.08  *p* = .927 | *F*(2, 537) = 0.32  *p* = .725 | *F*(2, 384) = 9.17  *p* < .001* | *F*(2, 639) = 16.03 *p* < .001* | *F*(2, 589)=11.26 *p* < .001* |

**Table 26** | Statistical values for main effects of Vignette and interaction effects between Vignette and Condition for each item. Significant *p*-values are indicated with “*”.

|  | Main Effect of Vignette | Vignette x Condition Interaction | Significant Pairwise Comparisons |
| --- | --- | --- | --- |
|  |  |  |  |
|  |  |  |  |
| Prosocial Behavior | *F*(2, 1607) = 1.60  *p* = .202 | *F*(4, 1607) = 0.11  *p* = .980 | -- |
|  |  |  |  |
| Empathy | *F*(2, 1606) = 0.35  *p* = .703 | *F*(4, 1606) = 1.06  *p* = .373 | -- |
|  |  |  |  |
| Trust | *F*(2, 1610) = 5.23  *p* = .005* | *F*(4, 1610) = 0.72  *p* = .581 | -- |
|  |  |  |  |
| Avoidance | *F*(2, 1607) = 0.40  *p* = .669 | *F*(4, 1607) = 0.31  *p* = .870 | -- |
|  |  |  |  |
| Willingness to Gossip | *F*(2, 1609) = 0.42  *p* = .655 | *F*(4, 1609) = 0.65  *p* = .626 | -- |
|  |  |  |  |
| Pursuit of Revenge | *F*(2, 1604) = 1.45  *p* = .234 | *F*(4, 1604) = 1.45  *p* = .214 | -- |
|  |  |  |  |
| Offender Recidivism | *F*(2, 1610) = 1.82  *p* = .163 | *F*(4, 1610) = 5.34  *p* < .001* | Punish Bike vs. Clay (*p* = .009*)  Punish Bike vs. Drawing (*p* = .002*) |
|  |  |  |  |
| Victim Positive Affect | *F*(2, 1603) = 0.89  *p* = .412 | *F*(4, 1603) = 0.89  *p* = .467 | -- |
|  |  |  |  |
| Offender Positive Affect | *F*(2, 1604) = 1.49  *p* = .225 | *F*(4, 1604) = 0.64  *p* = .635 | -- |
|  |  |  |  |
| Bystander Positive Affect | *F*(2, 1606) = 1.64  *p* = .195 | *F*(4, 1604) = 1.86  *p* = .010* | Do Nothing Bike vs. Clay (*p* = .011*) |
|  |  |  |  |
| Others’ Interest in Affiliating with Victim | *F*(2, 1604) = 0.47  *p* = .628 | *F*(4, 1604) = 1.07  *p* = .369 | -- |
|  |  |  |  |
| Others’ Interest in Affiliating with Offender | *F*(2, 1612) = 1.98  *p* = .139 | *F*(4, 1612) = 0.11  *p* = .978 | -- |

# **Table S27.** Word frequency for children’s, adolescents’, and adults’ free-response answers to the question, “Can you tell me what you think the word ‘punish’ means?”.

| **Children** |  | **Adolescents** |  | **Adults** |  |
| --- | --- | --- | --- | --- | --- |
| Frequency | Word | Frequency | Word | Frequency | Word |
| 85 | something | 70 | something | 88 | someone |
| 46 | bad | 66 | someone | 73 | something |
| 39 | someone | 36 | means | 42 | means |
| 17 | person | 32 | bad | 40 | punish |
| 16 | back | 32 | punish | 34 | person |
| 12 | mean | 24 | discipline | 26 | negative |
| 12 | punish | 22 | trouble | 23 | wrong |
| 11 | means | 22 | back | 20 | bad |
| 11 | know | 19 | person | 18 | back |
| 10 | trouble | 15 | give | 17 | pay |
| 8 | somebody | 15 | think | 16 | actions |
| 7 | thing | 14 | punishment | 15 | behavior |
| 7 | give | 14 | revenge | 15 | action |
| 6 | punished | 14 | actions | 14 | inflict |
| 6 | tell | 13 | hurt | 14 | hurt |
| 6 | time | 9 | consequence | 12 | harm |
| 5 | want | 8 | lesson | 12 | consequences |
| 5 | say | 8 | negative | 12 | done |
|  |  | 8 | away | 11 | punishment |
|  |  | 8 | behavior | 11 | give |
|  |  | 8 | wrong | 10 | penalty |
|  |  | 7 | consequences | 10 | retribution |
|  |  | 7 | word | 9 | revenge |
|  |  | 7 | penalty | 9 | consequence |
|  |  | 5 | getting | 9 | even |
|  |  | 5 | done | 8 | pain |
|  |  | 5 | teach | 8 | mean |
|  |  | 5 | pay | 8 | response |
|  |  | 5 | hit | 8 | things |
|  |  | 5 | thing | 7 | cause |
|  |  |  |  | 7 | teach |
|  |  |  |  | 7 | away |
|  |  |  |  | 7 | hold |
|  |  |  |  | 7 | accountable |
|  |  |  |  | 7 | physical |
|  |  |  |  | 7 | one |
|  |  |  |  | 6 | somebody |
|  |  |  |  | 6 | act |
|  |  |  |  | 6 | future |
|  |  |  |  | 6 | discipline |
|  |  |  |  | 6 | suffering |
|  |  |  |  | 6 | know |
|  |  |  |  | 6 | think |
|  |  |  |  | 6 | making |
|  |  |  |  | 6 | retaliate |
|  |  |  |  | 5 | feel |
|  |  |  |  | 5 | lesson |
|  |  |  |  | 5 | caused |
|  |  |  |  | 5 | suffer |
|  |  |  |  | 5 | committed |
|  |  |  |  | 5 | wrongdoing |
|  |  |  |  | 5 | meant |
|  |  |  |  | 5 | equal |
|  |  |  |  | 5 | retaliation |

# **Table S28.** Word frequency for children’s, adolescents’, and adults’ free-response answers to the question, “Can you tell me what you think the word ‘forgive’ means?”.

| **Children** |  | **Adolescents** |  | **Adults** |  |
| --- | --- | --- | --- | --- | --- |
| Frequency | Word | Frequency | Word | Frequency | Word |
| 43 | someone | 69 | someone | 67 | someone |
| 36 | forgive | 48 | forgive | 62 | let |
| 36 | something | 37 | means | 55 | go |
| 30 | sorry | 36 | something | 48 | person |
| 27 | bad | 35 | accept | 44 | means |
| 23 | say | 30 | person | 37 | hold |
| 21 | okay | 25 | say | 32 | something |
| 17 | person | 22 | okay | 27 | forgive |
| 12 | mean | 21 | sorry | 23 | feelings |
| 9 | just | 16 | mad | 21 | bad |
| 8 | want | 16 | let | 21 | move |
| 8 | mad | 16 | apology | 16 | past |
| 7 | tell | 15 | bad | 15 | angry |
| 7 | saying | 14 | think | 14 | done |
| 7 | um | 14 | go | 14 | wrong |
| 6 | nice | 14 | forget | 14 | anger |
| 5 | says | 14 | give | 14 | forgiveness |
| 5 | forgives | 10 | stop | 12 | negative |
| 5 | know | 10 | chance | 12 | grudge |
| 5 | accept | 9 | anymore | 12 | resentment |
| 5 | punishing | 9 | past | 11 | happened |
|  |  | 9 | feeling | 11 | ill |
|  |  | 9 | actions | 11 | towards |
|  |  | 9 | done | 11 | accept |
|  |  | 8 | trust | 10 | anymore |
|  |  | 8 | someone's | 10 | mean |
|  |  | 8 | move | 10 | incident |
|  |  | 8 | happened | 9 | longer |
|  |  | 8 | angry | 9 | toward |
|  |  | 7 | mistake | 8 | stop |
|  |  | 7 | saying | 8 | letting |
|  |  | 7 | grudge | 8 | committed |
|  |  | 7 | forgiving | 8 | release |
|  |  | 6 | back | 8 | tell |
|  |  | 6 | people | 8 | situation |
|  |  | 6 | wrong | 7 | punish |
|  |  | 5 | friends | 7 | actions |
|  |  | 5 | hold | 7 | put |
|  |  |  |  | 7 | action |
|  |  |  |  | 7 | without |
|  |  |  |  | 6 | know |
|  |  |  |  | 6 | feeling |
|  |  |  |  | 6 | understand |
|  |  |  |  | 6 | revenge |
|  |  |  |  | 6 | wronged |
|  |  |  |  | 5 | behavior |
|  |  |  |  | 5 | decide |
|  |  |  |  | 5 | understanding |
|  |  |  |  | 5 | mad |
|  |  |  |  | 5 | things |
|  |  |  |  | 5 | apology |
|  |  |  |  | 5 | accepting |
|  |  |  |  | 5 | wrongdoing |
|  |  |  |  | 5 | retribution |
|  |  |  |  | 5 | hard |
|  |  |  |  | 5 | holding |

**Figure S1.** Participants’ ratings as a function of Condition (punishment, do nothing, forgiveness) (on the left) and as a function of Age Group (children, adolescents, adults) and Condition (punishment, do nothing, forgiveness) (on the right) for the three items included in the “Prosocial Thoughts and Emotions” category. Error bars represent +/- confidence intervals.


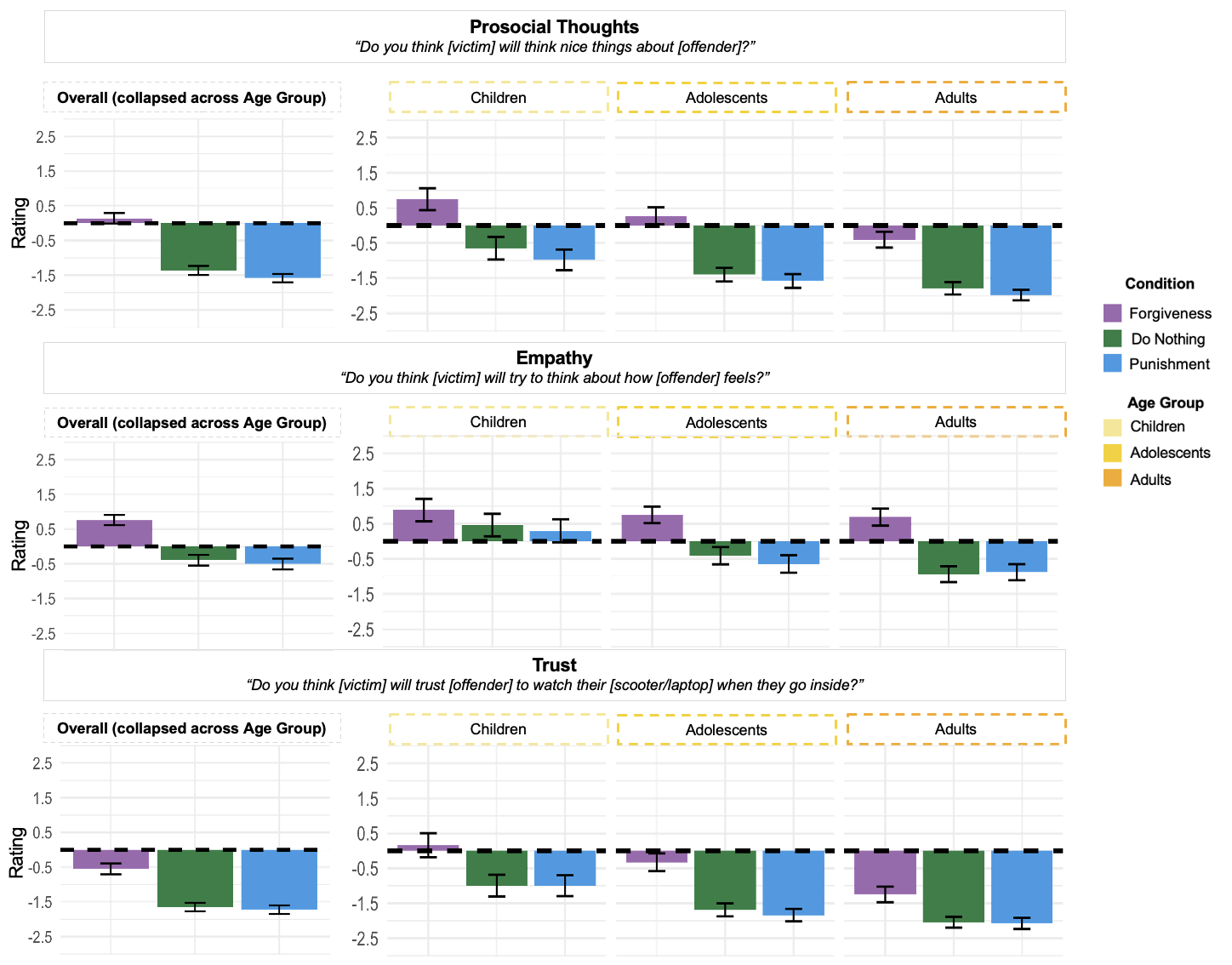


**Figure S2.** Participants’ ratings as a function of Condition (punishment, do nothing, forgiveness) (on the left) and as a function of Age Group (children, adolescents, adults) and Condition (punishment, do nothing, forgiveness) (on the right) for the three items included in the “Antisocial Behaviors” category. Error bars represent +/- confidence intervals.

**
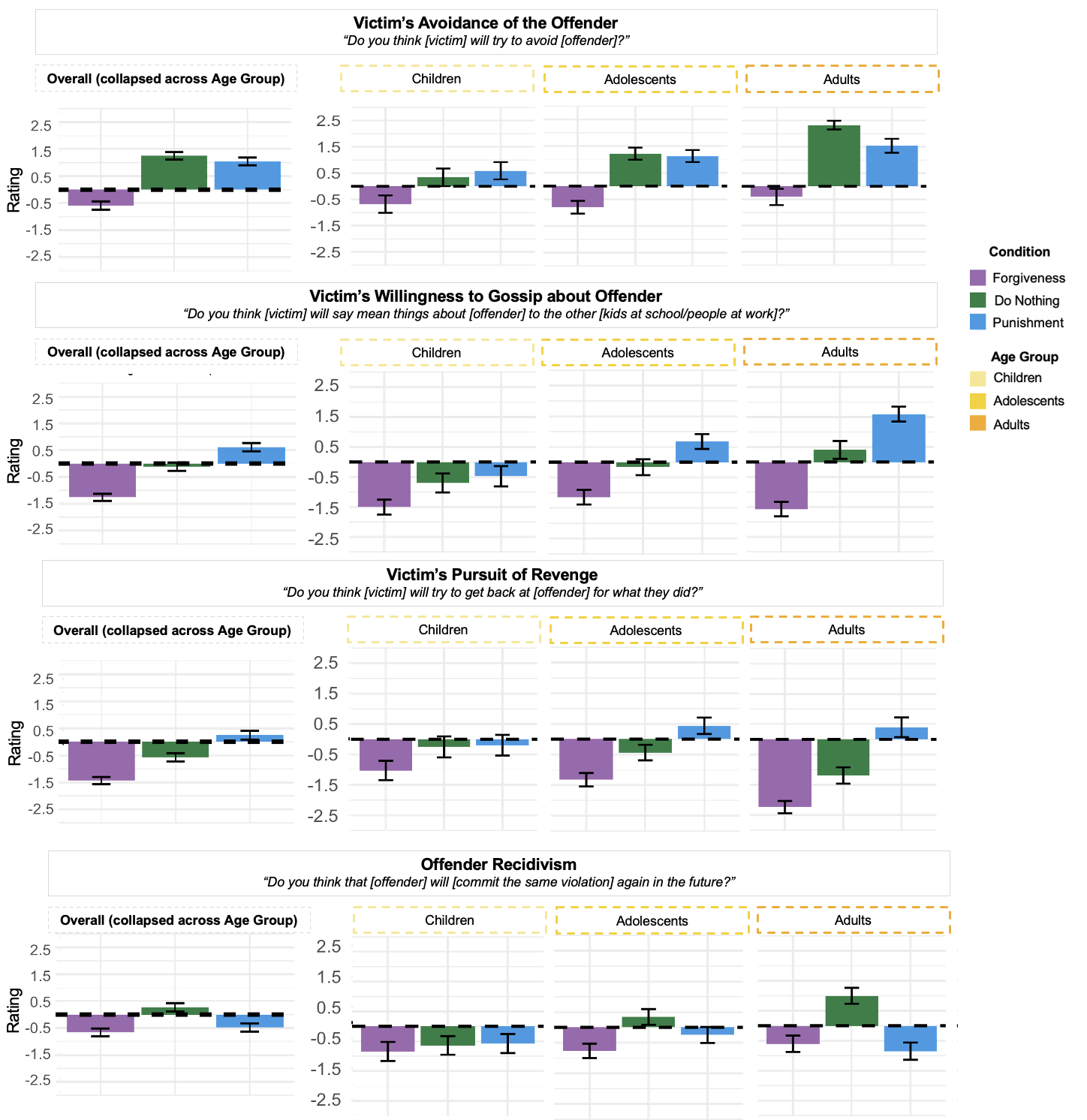
**

**Figure S3.** Participants’ ratings as a function of Condition (punishment, do nothing, forgiveness) (on the left) and as a function of Age Group (children, adolescents, adults) and Condition (punishment, do nothing, forgiveness) (on the right) for the three items included in the “Affective Change” category. Error bars represent +/- confidence intervals.


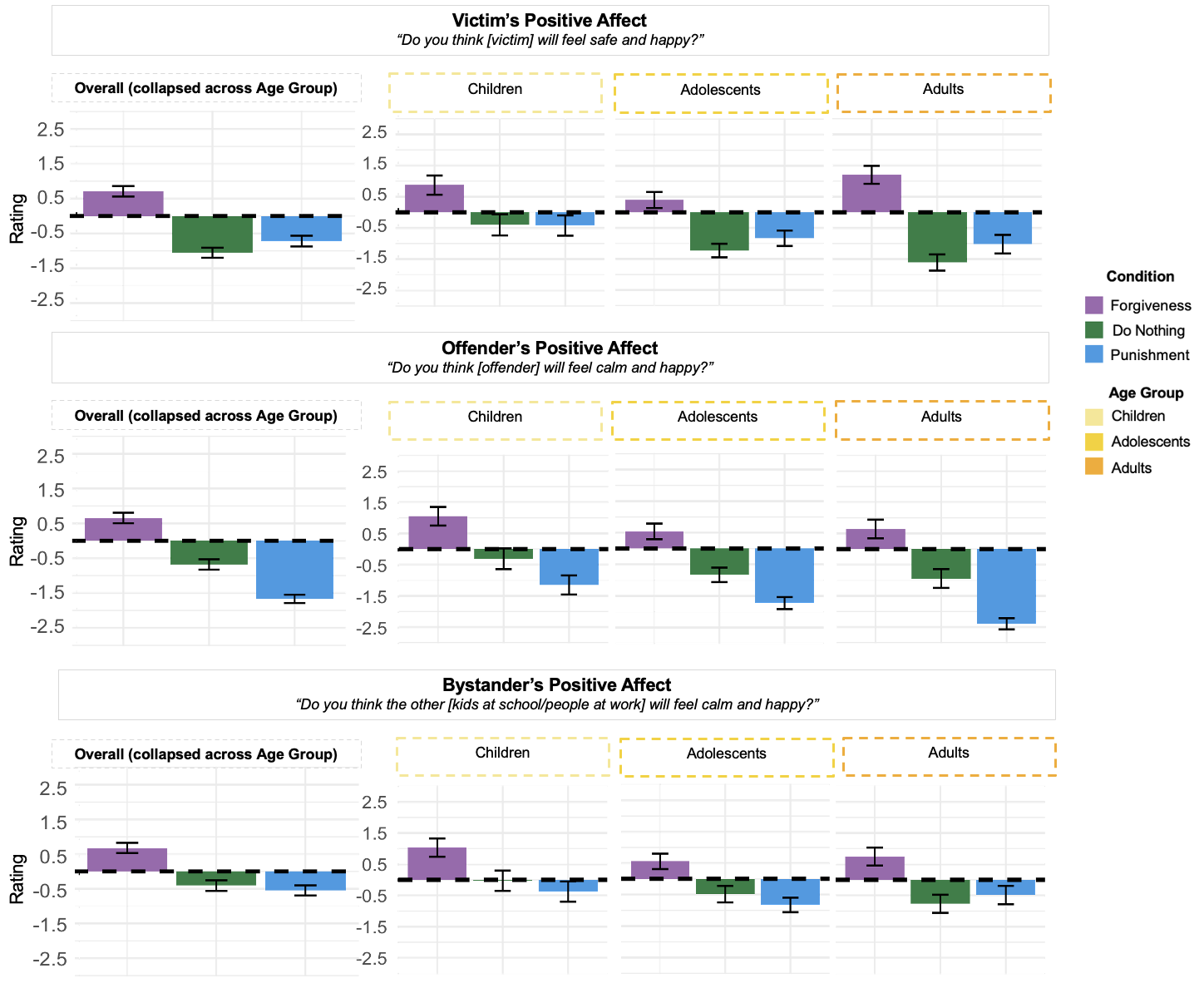


# **Figure S4.** Participants’ ratings as a function of Condition (punishment, do nothing, forgiveness) (on the left) and as a function of Age Group (children, adolescents, adults) and Condition (punishment, do nothing, forgiveness) (on the right) for the three items included in the “Affiliative Interest” category. Error bars represent +/- confidence intervals.


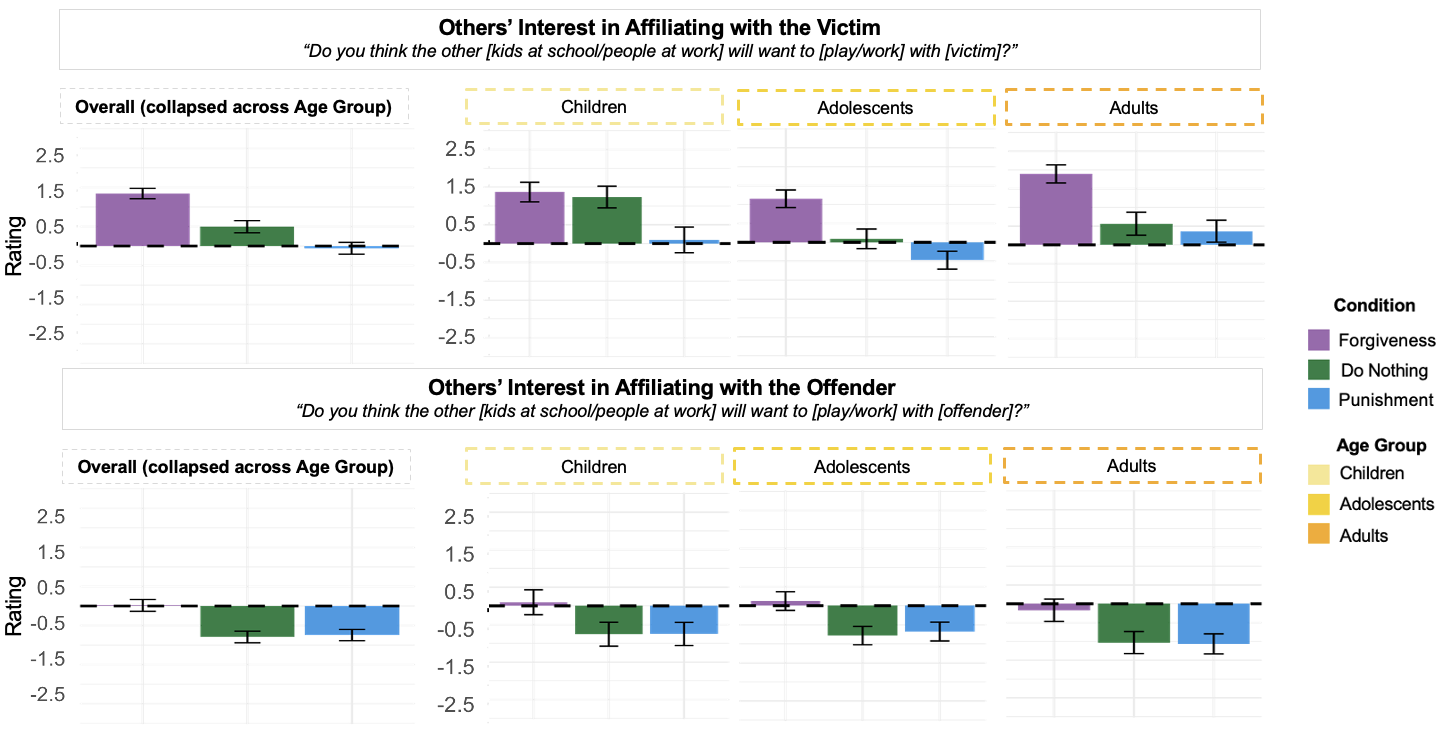


# **Figure S5.** Participants’ ratings as a function of Condition (punishment, do nothing, forgiveness) and Vignette (Bike, Clay, Drawing) for Victim Prosocial Behavior.


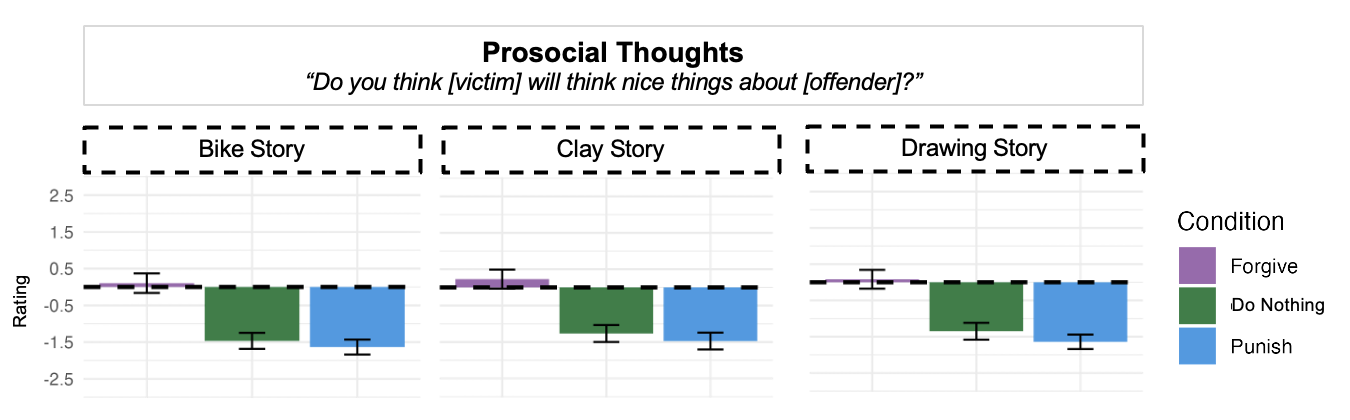


# **Figure S6.** Participants’ ratings as a function of Condition (punishment, do nothing, forgiveness) and Vignette (Bike, Clay, Drawing) for Victim’s Empathy for the Offender.


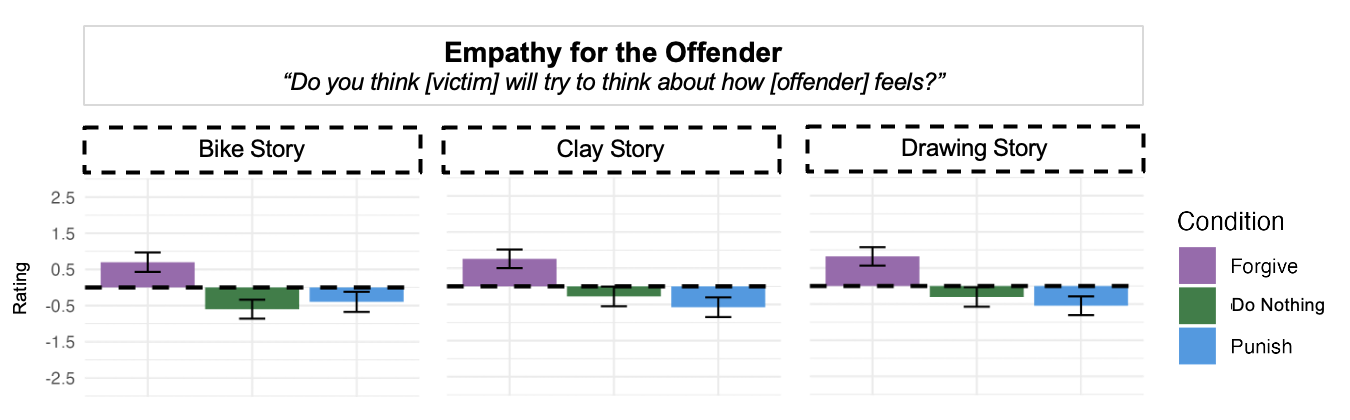


# **Figure S7.** Participants’ ratings as a function of Condition (punishment, do nothing, forgiveness) and Vignette (Bike, Clay, Drawing) for Victim’s Trust of the Offender.


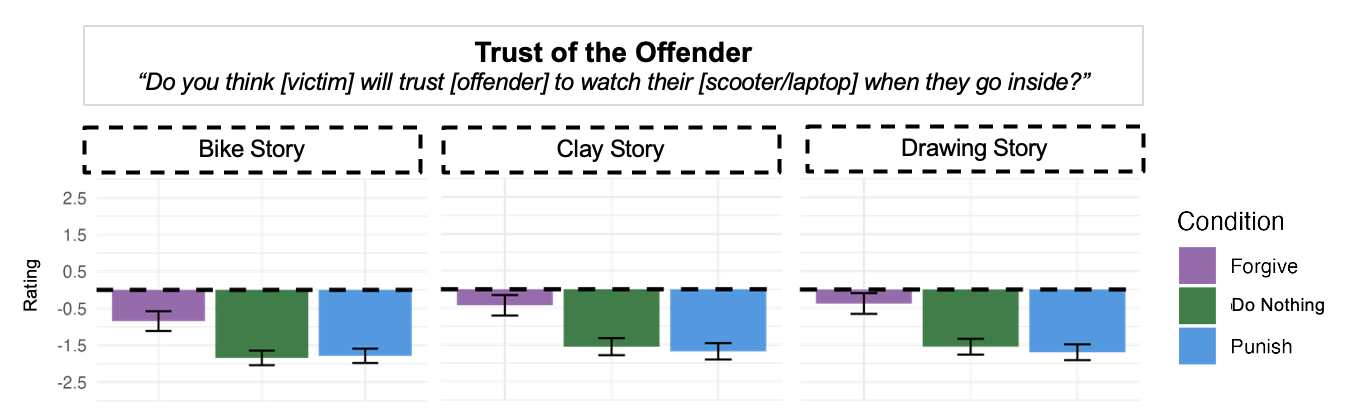


# **Figure S8.** Participants’ ratings as a function of Condition (punishment, do nothing, forgiveness) and Vignette (Bike, Clay, Drawing) for Victim’s Avoidance of the Offender.


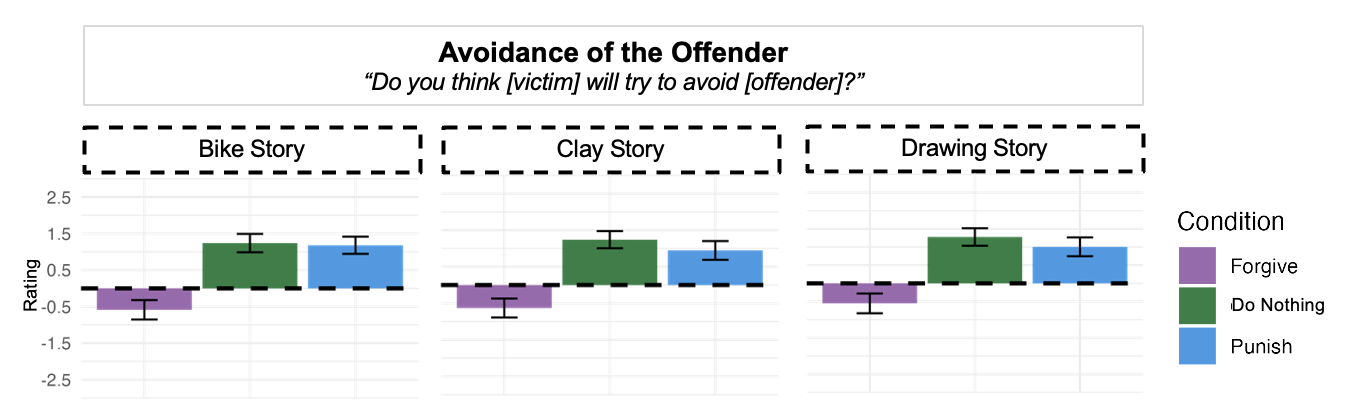


# **Figure S9.** Participants’ ratings as a function of Condition (punishment, do nothing, forgiveness) and Vignette (Bike, Clay, Drawing) for Victim’s Willingness to Gossip About the Offender.


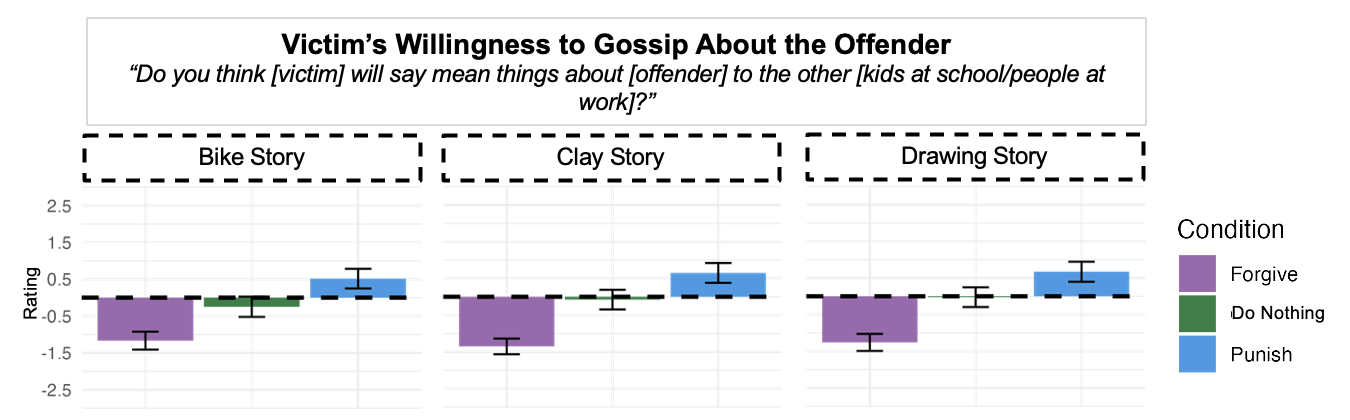


# **Figure S10.** Participants’ ratings as a function of Condition (punishment, do nothing, forgiveness) and Vignette (Bike, Clay, Drawing) for Victim’s Pursuit of Revenge.


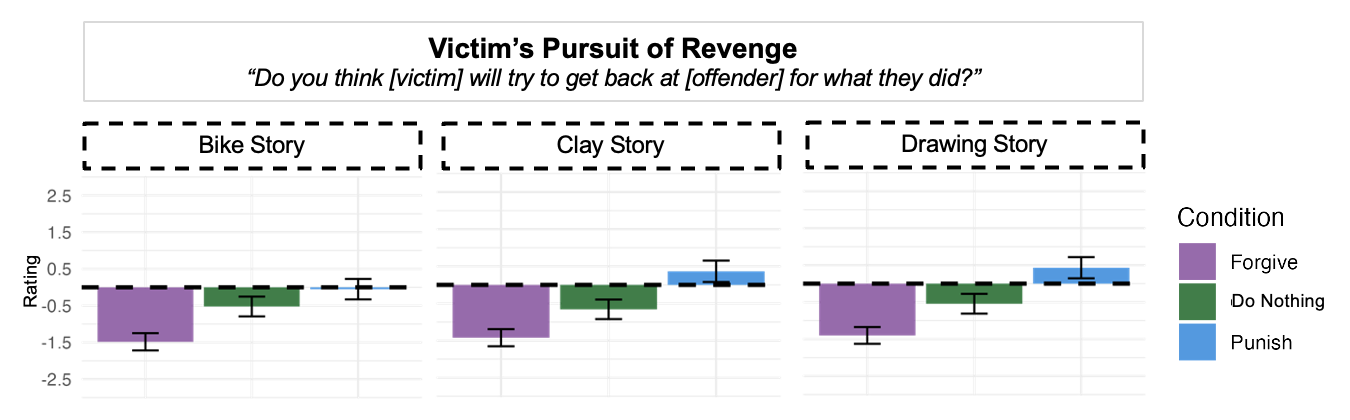


# **Figure S11.** Participants’ ratings as a function of Condition (punishment, do nothing, forgiveness) and Vignette (Bike, Clay, Drawing) for Offender Recidivism.


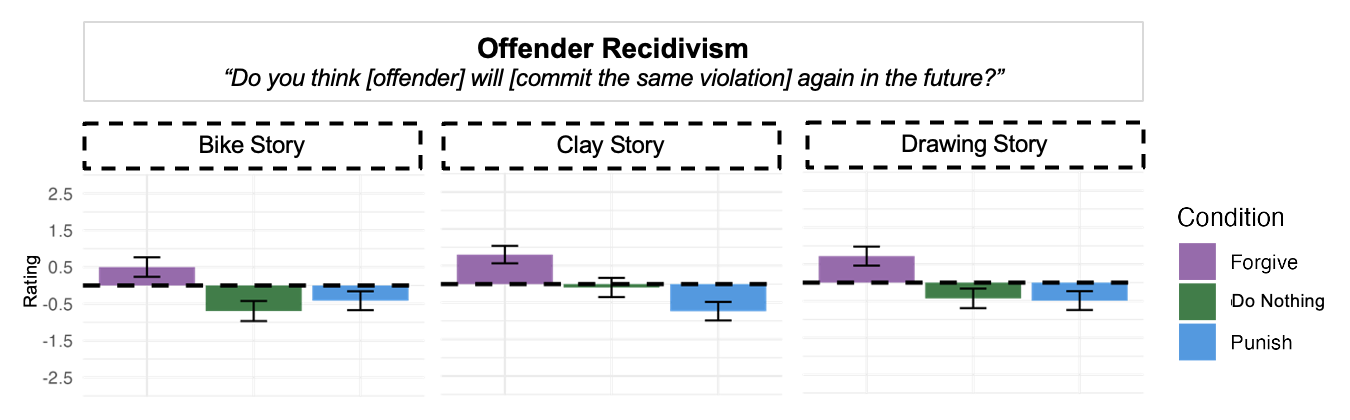


# **Figure S12.** Participants’ ratings as a function of Condition (punishment, do nothing, forgiveness) and Vignette (Bike, Clay, Drawing) for Victim Affective Change.


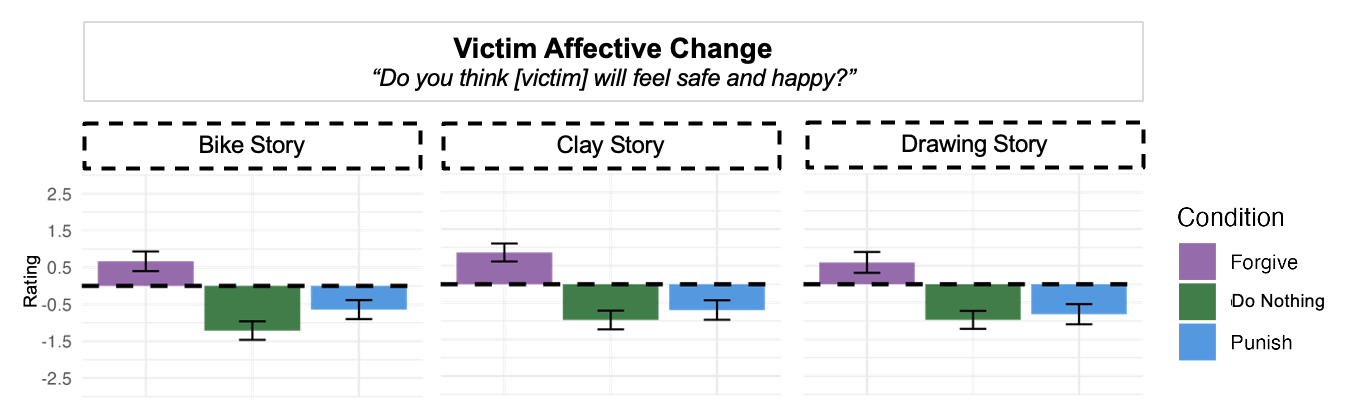


# **Figure S13.** Participants’ ratings as a function of Condition (punishment, do nothing, forgiveness) and Vignette (Bike, Clay, Drawing) for Offender Affective Change.


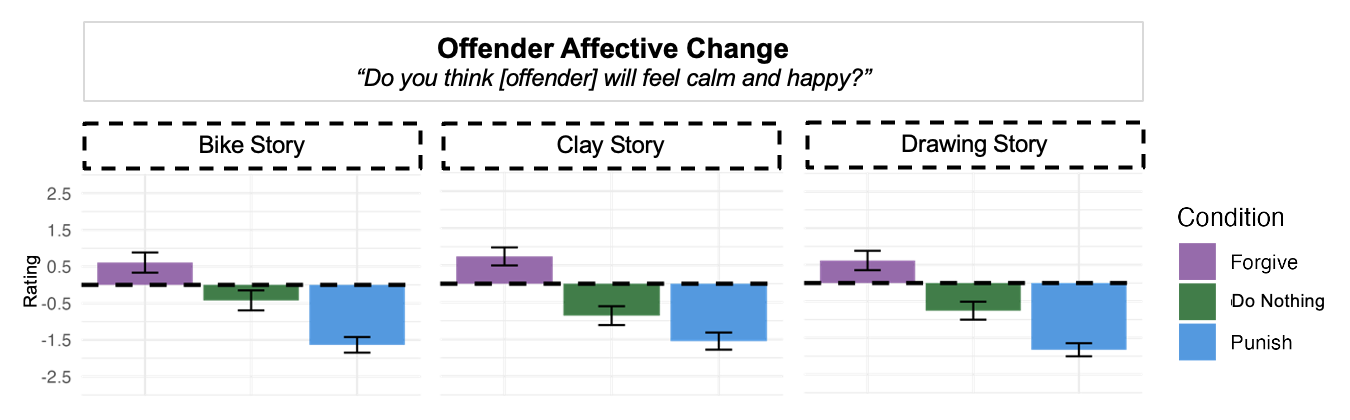


# **Figure S14.** Participants’ ratings as a function of Condition (punishment, do nothing, forgiveness) and Vignette (Bike, Clay, Drawing) for Bystander Affective Change.


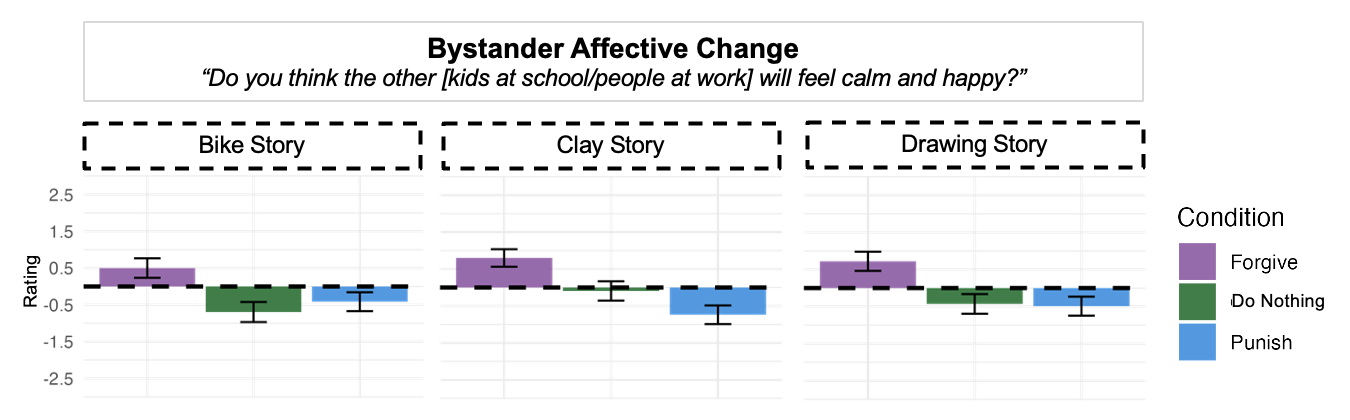


# **Figure S15.** Participants’ ratings as a function of Condition (punishment, do nothing, forgiveness) and Vignette (Bike, Clay, Drawing) for Others’ Evaluation of the Victim.


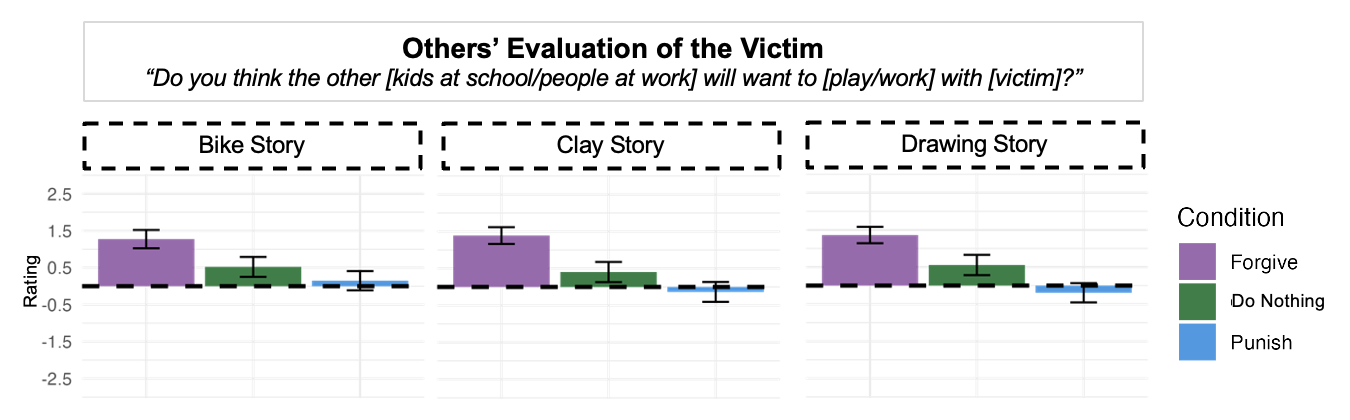


# **Figure S16.** Participants’ ratings as a function of Condition (punishment, do nothing, forgiveness) and Vignette (Bike, Clay, Drawing) for Others’ Evaluation of the Offender.


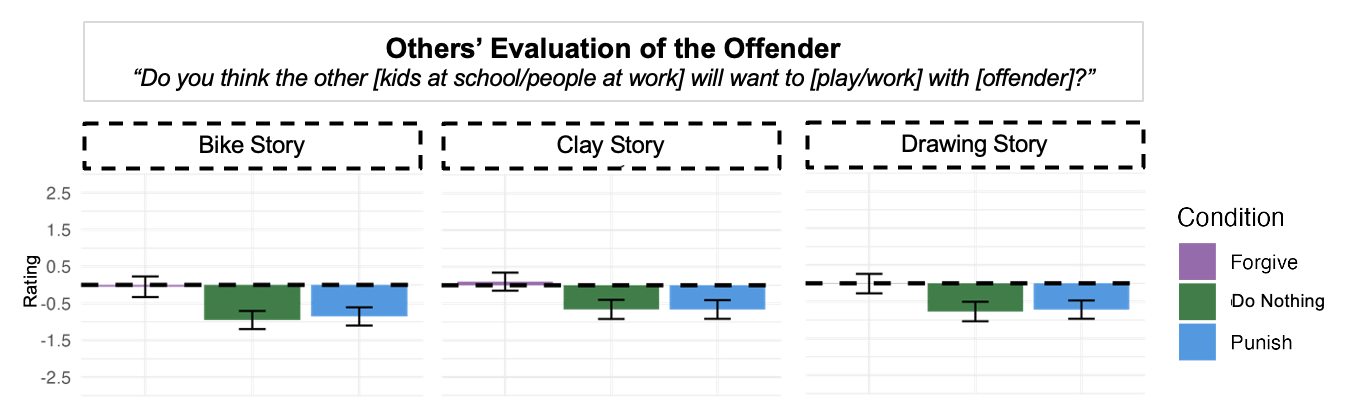

Supplement: Supplementary file 1 — Appendix S1. [file CDEV-95-1915-s001.docx]
